# Isolation and characterization of lytic bacteriophages from various sources in Addis Ababa against antimicrobial-resistant diarrheagenic *Escherichia coli* strains and evaluation of their therapeutic potential

**DOI:** 10.1186/s12879-024-09152-z

**Published:** 2024-03-14

**Authors:** Tamirat Salile Sada, Tesfaye Sisay Tessema

**Affiliations:** 1 Institute of Biotechnology, Addis Ababa University, P.O.Box 1176, Addis Ababa, Ethiopia; 2 Department of Biotechnology, Woldia University, P.O. Box 400, Woldia, Ethiopia

**Keywords:** Lytic bacteriophages, Diarrheagenic *E. coli*, *Myoviridae*, *Siphoviridae*, *Podoviridae*

## Abstract

**Background:**

*Escherichia coli* is a common fecal coliform, facultative aerobic, gram-negative bacterium. Pathogenic strains of such microbes have evolved to cause diarrhea, urinary tract infections, and septicemias. The emergence of antibiotic resistance urged the identification of an alternative strategy. The use of lytic bacteriophages against the control of pathogenic *E. coli* in clinics and different environmental setups (waste and drink water management) has become an alternative therapy to antibiotic therapy. Thus, this study aimed to isolate and characterize lytic bacteriophage from various sources in Addis Ababa, tested them against antimicrobial-resistant diarrheagenic *E. coli* strains and evaluated their therapeutic potential under in vitro conditions.

**Methods:**

A total of 14 samples were processed against six different diarrheagenic *E. coli* strains. The conventional culture and plaque analysis agar overlay method was used to recover lytic bacteriophage isolates. The phage isolates were characterized to determine their lytic effect, growth characteristics, host range activity, and stability under different temperature and pH conditions. Phage isolates were identified by scanning electron microscope (SEM), and molecular techniques (PCR).

**Results:**

In total, 17 phages were recovered from 84 tested plates. Of the 17 phage isolates, 11 (65%) were *Myoviridae*-like phages, and 6 (35%) phage isolates were *Podoviridae* and *Siphoviridae* by morphology and PCR identification. Based on the host range test, growth characteristics, and stability test 7 potent phages were selected. These phages demonstrated better growth characteristics, including short latent periods, highest burst sizes, and wider host ranges, as well as thermal stability and the ability to survive in a wide range of pH levels.

**Conclusions:**

The promising effect of the phages isolated in this study against AMR pathogenic *E. coli* has raised the possibility of their use in the future treatment of *E. coli* infections.

**Supplementary Information:**

The online version contains supplementary material available at 10.1186/s12879-024-09152-z.

## Background

*E. coli* is a prominent fecal coliform facultative aerobic gram-negative rod-shaped bacterium. Pathogenic strains have evolved to cause human and animal diseases leading to human and animal morbidity and mortality worldwide. Furthermore, the globalization of food marketing and distribution exacerbates the risk of sickness connected with this pathogenic *E. coli*. Both developed and developing nations frequently experience outbreaks, which can occasionally have fatal consequences [1].

Even though the majority of *E. coli* are thought to be relatively harmless and are part of the normal flora of the intestine, certain strains have evolved pathogenicity mechanisms and can cause diseases in humans and animals. Intestinal (diarrhea) and extraintestinal (urinary tract infection (UTI), septicemia, pneumonia, and meningitis) disorders are examples [2].

There are seven diarrheagenic *E. coli* serotypes including enteropathogenic *E. coli* (EPEC), enterohaemorrhagic *E. coli* (EHEC/O157), enteroinvasive *E. coli* (EIEC), enteroaggregative *E. coli* (EAEC), enterotoxigenic *E. coli* (ETEC), diffusely adherent *E. coli* (DAEC) and Shiga toxin-generating *E. coli* (Non-O157 STEC). These have been identified as *E. coli* pathotypes based on pathogenicity profile, virulence factors, clinical disease, and phylogenetic profiles. Adherent invasive *E. coli* (AIEC), which is typically linked to inflammatory bowel disease (IBD) has recently evolved [3–5].

Antibiotic treatments for *E. coli* frequently result in the spread of multidrug resistance (MDR) in clinics. Antibiotics are becoming increasingly less efficient as pathogenic *E. coli* develop resistance to them as a result of widespread clinical, veterinary, and agricultural use [6]. There are growing reviews of *E. coli* AMR worldwide. Because these organisms are naturally found in high concentrations in human and animal feces, when the feces are disposed of and reach drainage systems, where already overused or misused antibiotics released from clinical aspects and agricultural run-offs predominate, coliforms are under pressure and AMR strains emerge [7, 8].

The persistence of AMR microbes not only puts selective pressure on nearby exposed bacteria, but also increases opportunities for resistance genes to be transferred to nearby susceptible bacteria (via horizontal gene transfer using plasmids, transposons, or integrons) and eventually into the human food chain [9]. This allows them to thrive in the absence of inhibition or toxicity.

The spread of multidrug resistance among bacterial strains has posed a substantial threat to public health in the treatment of infectious diseases on a global scale [10]. Although there is a high global rise in antibiotic-resistant bacteria, the search for new antibiotics has slowed in recent decades [11]. As a result, continuous efforts to discover promising alternative therapies for the treatment of infectious diseases and proscribe the emergence and unfolding of antibiotic resistance among microorganisms are needed [9]. In this regard, scientists’ interest in phages as an alternative medicine was rekindled. The use of lytic bacteriophages as a means of combating pathogenic bacterial contamination is an alternate technique [12]. Phage therapy or the use of bacteriophages as therapeutic agents for eradicating bacterial infections was first introduced by a preliminary study by Twort and d’Herelle in the beginning of the twentieth century [13, 14].

Phage therapy is the therapeutic application of bacteriophages (natural bacteria predators) to treat bacterial illnesses. Phage therapy has regained popularity as an alternative to antibiotics. Natural phage communities are presumed to be reservoirs of significant uncharacterized genetic variation on Earth [15]. Complete phage genomes make it easier to research phage evolution, relationships, biodiversity, biogeography, and the discovery of new phage taxa [7]. Understanding phage biology can lead to a wide range of applications, including innovative nanotechnologies, bacterial detection techniques, and industrial-scale biological control of harmful bacteria.

Individual phages or a cocktail of phages are often used in therapeutic techniques to specifically infect and kill target microorganisms. Bacteriophages have the ability to influence pathogenic bacteria without inflicting collateral damage to the commensal microbiota due to their limited species-specific host range [16–18].

Furthermore, phages are the most “safe” and “green” creatures that can be used in clinical settings [19, 20]. Apart from their ability to self-replicate and destroy antibiotic-resistant bacteria, they are abundant in nature and have excellent selectivity, causing minimal damage to regular flora [21]. Unlike antibiotics, which lose potency over time after administration, phages continue to reproduce and infect target bacteria [10]. When compared to antibiotics, phage resistance is not transferable. In addition, compared to the development of a new antibiotic, the isolation of a new phage is comparatively quick and inexpensive. The use of bacteriophages as antimicrobial agents necessitates a thorough understanding of phage biology to assess their potential as an alternative effective technique for the control of pathogenic bacteria [22, 23].

Over 95% of the lytic phages reported in the scientific literature belong to the order *Caudovirales* (tailed phages) [24, 25] that have double-stranded linear DNA. This order mainly includes the *myoviridae* (T4-like phages), *podoviridae* (T7-like phages), and *siphoviridae* (T5-like phages) families of bacteriophages that are actively involved in the lysis and destruction of bacteria. However, lytic phages are not limited to the order C*audovirales*, and many bacteriophages exist on Earth, such as single-stranded linear RNA MS2 phages as well as many others that are mostly temperate or lysogenic [26].

Despite the growing threat of antimicrobial resistance in our country, little attention is paid to its management, and novel alternatives have yet to be explored. Furthermore, as a biological resource-rich country with a diverse range of environmental elements, investigating lytic phages in our capital city environment could be a potential solution to combating antibiotic resistance. However, not much study has been done on phage isolation generally from various environmental samples in Ethiopia and Addis Ababa in particular, as well as the testing of these phages against pathogenic and drug-resistant *E. coli* strains. There is limited study on the individual bacteriophages that infect *E. coli* strains.

Thus, the present study was carried out to isolate, characterize, and examine the therapeutic potential of pure phage strains against diarrheagenic *E. coli* strains from various sources in Addis Ababa, Ethiopia.

## Methods

### Description of the study area and period

**Fig. 1:**
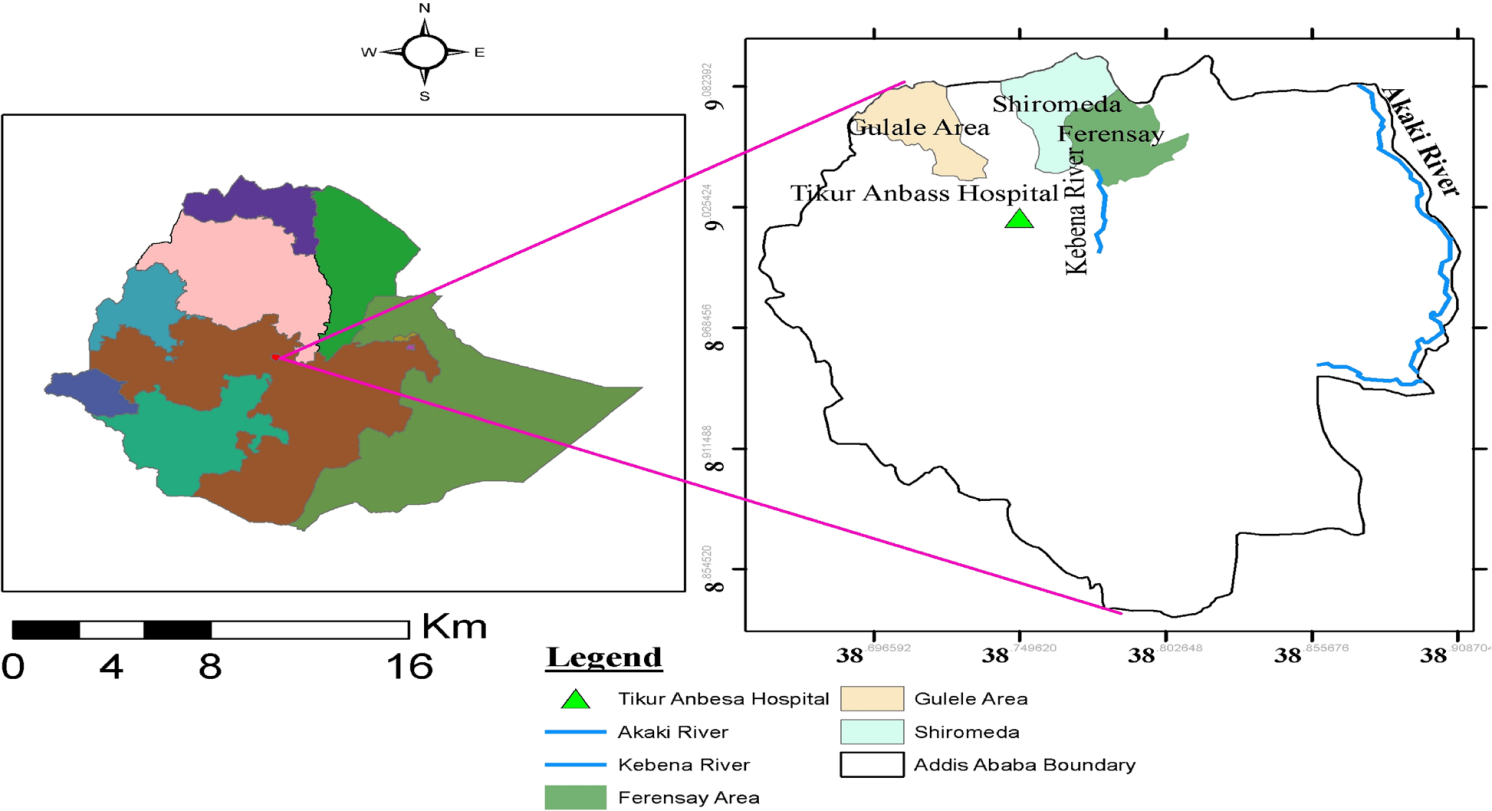
Study area geographic map

The study was conducted in selected areas of Addis Ababa namely, the Kebena River, Akaki River, dairy farm sewages in Gulele, Shiromeda, and Ferensay areas in the city and Tikur Anbessa Hospital fluid waste disposal. Samples were processed at the health biotechnology laboratory, Institute of Biotechnology, Addis Ababa University from April 2022 to May 2023. Ethiopia’s largest city and capital is Addis Ababa. Depending on elevation and predominant wind patterns, the city has a complex mix of highland climate zones with temperature variations of up to 10 °C (50 ° F). Addis Ababa has DMS latitude longitude coordinates of 9° 0′ 19.4436″ N, 38° 45′ 48.9996″ E (Fig. 1). The high elevation moderates’ temperatures year-round and the city’s position near the equator means that temperatures are very constant from month to month. The temperature in January ranges from 20 °C (68 °F) to 12 °C (53 °F). The area of the city increased from 85.73 mile^2^ (222.04 km^2^) in 1984 to 204.7 mile^2^ (530.21 km^2^) in 1994 to 2023. Addis Ababa had a population of 5,461,000 and a 4.46% annual growth rate in 2023 according to Macrotrends, Metro area population [27].

### Study design and sampling method

A descriptive study design was employed for the isolation and characterization of potent *E. coli* lytic phages present in the sampling sites. A convenient nonprobability purposive sampling was used for the selection of sampling sites and sample size determinations. Thus, a total of 14 samples, including 3 from Kebena River (top, middle, and bottom surface of the collection pond), 3 from Akaki River (top, middle, and bottom surface of the collection pond), 6 from dairy sewage from the Gulele, Shiromeda and Ferensay areas (superficial and stirred sediment) and 2 from Tikur Anbessa hospital waste samples (superficial and stirred sediment) were tested against the six *E. coli* strains.

### Sample collection and processing

Fifty milliliters of samples were collected from the stagnant surface of river water from 3 regions (top (5 cm), middle (25 cm) and bottom (50 cm)) while in the case of hospital effluent and dairy farm effluent, samples were collected from the superficial and stirred sediment of selected site in a 50 ml sterile screw-capped glass bottle. In total, 14 samples were collected from the selected sites; water (3 from Kebena and 3 from Akaki river effluents, 2 from Gulele dairy farms, 2 from Shiromeda dairy farms, 2 from Ferensay dairy farms and 2 from Tikur Anbessa hospital waste) samples.

After collection, the samples were transported to the lab in an ice box and stored overnight at room temperature to settle large debris and insoluble waste. The remaining particulates were removed by centrifugation at 3000 rpm for 10 min using 15 ml Falcon tubes, and the supernatant was slowly filtered through a syringe filter with a pore size of 0.22 μm to a sterile screw-capped tube.

### Bacterial host strains and culture conditions

The bacterial host cells used in this study include a total of six clinically isolated pathogenic *E. coli* strains, shown in Table 1, that are found as culture collections at the laboratory of Prof. Tesfaye Sisay Tessema, Institute of Biotechnology, Addis Ababa University. The bacterial isolates were stored as glycerol stocks at − 20 °C. The *E. coli* isolates were grown at 37 °C in Eosin methylene blue, tryptone soy broth and soft agar overlays (0.75% agar).

**Table 1:** *E. coli* strains used as host and host range tests

No	Isolate code	Strains	Antibiotics profile	Source
1	EHEC-52A	Enterohemorrhagic *E. coli* (EHEC)	Resistant to CMP, GM, AMX	Children, diarrheic
2	EPEC-46A	Enteropathogenic *E. coli (*EPEC)	Resistant to AMP, AMX, NOR, CS3	Childern, diarrheic
3	STEC-29A	Shiga-toxin producing *E. coli* (STEC)	Resistant to TMT, CS3, AMP, CMP, AMX	Calf, diarrheic
4	ETEC-41A	Enterotoxigenic *E. coli* (ETEC)	Resistant to TMT, GM, AMX	Calf, diarrheic
5	EAEC-02A	Enteroaggregative *E. coli* (EAEC)	Resistant to CIP, AMP	Children, diarrheic
6	EIEC-24	Enteroinvasive *E. coli* (EIEC)	Resistant to CIP, TC, AMX, GC, AMP	Calf, diarrheic

*E. coli* strains resistant to three or more antibiotics were considered MDR strains

### Enrichment of the samples

The samples were purified through preliminary filtration using centrifugation and a 0.22 μm syringe filter, *E. coli* host strains were prepared in broth as an overnight culture. Then, 5 ml of tryptone soy broth culture for each sample was prepared by supplementing with 2 mM calcium chloride. Five ml of tryptone soy broth, 3 ml of each of the 6 *E. coli* strain broth cultures, and 3 ml of each sample were aseptically added to an appropriately labeled sterile 15 ml Falcon tube and incubated at 37 °C for 24 h at 150 rpm shaking. Following incubation, phage-infected cultures were poured into several 15 ml centrifuge tubes and centrifuged at 3000 rpm for 20 min to remove residue. The supernatant was decanted into a sterile tube and filtered through a syringe filter (0.22 μm) to remove bacterial cells.

### Bacteriophage isolation

The double-layer plaque technique was performed for bacteriophage isolation. Each of the 14 sample filtrates was individually tested in each of the 6 *E. coli* strains. Thus, 14*6 = 84 plates were tested for lytic phages. One milliliter of purified phage filtrate was transferred into a sterile test tube for each of the samples. Then, 2 ml of the respective *E. coli* tryptone soy broth grown culture at the log phase was added and mixed well. The mixture was left for 10 min at ambient temperature to allow phages to adsorb to the host. After 10 min, 5 mL tryptone soy agar (TSA) (0.7% agar) was added, mixed well, and poured over the surface of the tryptone agar plate. It was allowed to set at room temperature and incubated at 37 °C for 24 h. Plates were observed and scored as positive if there was a clear zone (plaque formation) over the surface of the agar plate. Plaques were counted from all positive samples and recorded as a plaque-forming unit (pfu/ml).

#### Purification of phages

Clear plaques were chosen and removed from the agar surface using sterile pipette tips to purify the phages. These plaques were then mixed with 1 ml of magnesium phage buffer (100 mM NaCl, 8 mM MgSO4, and 50 mM Tris-HCl (pH 7.5)) while being stirred in a vortex mixer. Centrifugation was used to remove the agar and cell remnants for 10 minutes at 3000 rpm. The supernatant was then filtered through a syringe filter with a pore size of 0.22 μm. The resultant filtrate, known as phage lysate, was stored at 4 °C until processing and utilized to evaluate the lytic efficacy of bacteriophages in vitro. To generate pure single plaques from each isolate, this process was performed three times [28, 29].

#### Titer determination of phages

Plaque-forming units/mL (PFU/mL), a measure of the bacteriophage concentration, was calculated by counting the number of plaques that developed in tryptone soy agar medium plates according to Sjahriani et al. (2021) with some modifications [29]. After serially diluting each lytic bacteriophage stock 10 times in sterile phage buffer solution, 100 μL of the bacteriophage isolate was added to 100 μL of the *E. coli* culture, which was cultured for 24 hours on tryptone soy broth medium. The suspension was combined with 5 mL of molten tryptone agar and incubated for 30 minutes at 37 °C. Following that, each suspension was put onto a TSA plate and left for 24 h at 37 °C in the incubator. Following incubation, plaque formation on the plates was monitored and the results were expressed as PFU/mL, which was calculated using the formula PFU/ml = (Plaques per plate) X (dilution factor)/(Volume of phage plated in ml). For the subsequent processes, a considerable plaque-forming sample material was selected.

#### Morphological imaging of phages

Scanning electron microscope (SEM) analysis was performed at Adama Science and Technology University, Department of Biology, Adama, Ethiopia. The liquid phage samples were lyophilized for SEM analysis using a lyophilizer machine (ALPHA 2–4 LD Plus, Christ) at the Microbial, Cellular and Molecular Biology Department, CNCS, Addis Ababa University. Gelatin and glucose were used as stabilizers of phage samples during lyophilization to protect phage degradation. For imaging SEM was operated between 5 and 15 kV accelerating voltage and focal depth between 5 and 50 μm with magnifications ranging from 1000X to 5000X.

### Characterization of selected phages

#### Determination of host range

Host range spectrum determination was performed using the spot method to check the ability of phages to lyse other bacteria in addition to their own host bacteria. The lytic spectra of a phage, which is a significant biological characteristic, refers to the range of bacteria genera, species, and strains that a phage can kill. The isolated bacteriophages were screened for their ability to infect bacterial cells to determine their infectivity range or lysis efficiency. This assay was based on the ability of phage to either produce a clear plaque, turbid plaque, or no lysis [30] against 6 *E. coli* strains and other bacterial species including *Shigella flexneri, Klebsiella pneumoniae* and *Salmonella* sp. Bacterial lawns of all the strains were prepared on tryptone soy agar and 10 μl droplets of phage lysates were spotted on these lawns using sterile pipette tips. The plates were incubated at 37 °C for 24 h and checked for the presence of plaques [7]. The most efficient phages based on the lysis profiles, plaque clarity, and sizes thus displaying zones of lysis against most of the isolates were selected for further studies.

### Efficiency of plating (EOP)

The efficiency of plating (EOP) was tested to measure the ability phages to form plaques on a bacterial lawn. It represents the ratio of the number of plaques formed by a particular phage on a host bacterial lawn to the number of phage particles applied to the lawn. The EOP analysis was performed on five bacterial strains, including *Klebsiella pneumoniae*, *Salmonella typhimurium*, *Shigella flexineri*, and STEC, that were susceptible to phages in the spot test [16].

#### Optimal multiplicity of infection (MOI)

The optimal MOI was determined as the ratio of the number of phages to that of host bacteria present in a defined space that is best for phage proliferation to obtain maximum titers. It represents the number of phage particles added per bacterial cell in a culture. *E. coli* cultures of the exponential growth phase were infected with different amounts of phages with a set of serial dilutions at 10 (10/1), 1 (1/1), 0.1 (1/10), 0.01(1/100), 0.001(1/1000), and 0.0001(1/10,000) [16, 31]. Once the phage was incubated for 2 hours, the titers were measured.

#### One-step growth curve of phages

The one-step growth curve was drawn using the MOI of phages for their isolation host. The growth curve of phage isolates was plotted over a 15 min to 60 min incubation time range, and the titer was calculated at each 15 min time interval using double layer agar assay. Then, the latent periods, relative burst size, and burst period were obtained from each graph for each phage isolate [32].

#### Temperature and pH stability test

The stability of the isolated pure bacteriophages at various pH and temperature levels was also determined. A 2.7 × 10^8^ to 1 × 10^9^ PFU/ml titer of phage lysate was evaluated using the double agar layer method after being stored at 25 °C, 37 °C, 70 °C, and 90 °C for 6 h to determine the stability of bacteriophages at various temperatures. Likewise, using double-layer agar plate techniques, phage stability at various pH values was assessed by culturing the phages in phage buffer at pH values of 3, 5, 7, 9, and 11 for 6 h [7, 33].

### Molecular identification of phages

#### DNA extraction and assessment quality

The organic DNA extraction method with subsequent ethanol precipitation was employed to retrieve DNA from bacteriophages. Bacteriophage DNA isolation was performed by adding 0.5 μL of DNaseI and 1 μL of RNase A to 1 mL of purified bacteriophage and incubating at 37 °C for 30 min to digest contaminating bacterial DNA and RNA. Phage nucleic acid was extracted by adding 40 μL of EDTA 0.5 M, 50 μL of 10% sodium dodecyl sulfate (SDS) and 5 μL of proteinase K (10 mg/ mL) and incubating the mixture at 37 °C for 1 h. After incubation, 700 μL of phenol–chloroform–isoamyl alcohol solution (25:24:1) was added to remove unwanted materials, and the solution was centrifuged at 13000 RPM for 5 min. Isopropyl alcohol was added to precipitate the DNA. Approximately 700 μL of 70% ethanol was added to the pellet, and the mixture was centrifuged again at 12,749×g for 10 min. The supernatant was removed and the pellet was dried. Fifty microliters of nuclease-free water (NFW) solution were added to the pellet for DNA storage at 4 °C [34]. In addition, to check the quality of phage DNA comparative DNA extraction was performed using DNase, RNase, and SDS and proteinase K treatment at different levels. That was phage filtrate with treatment of DNase and RNase and without treatment of DNase and RNase; phage-host mix with DNase, RNase, SDS, proteinase K treatment and without treatment. The comparative results were observed after 1% agarose gel electrophoresis.

Quantification and purity of the extracted DNA were estimated using a Nanodrop spectrophotometer. The quality concerning host DNA contamination was assessed using gel electrophoresis. The quantified and assessed DNA extract was prepared and used for PCR.

#### PCR and analysis of PCR products

The determination of lytic phage type was performed by conventional PCR using isolated phage DNA as a template and primers specific for the phage family and genus. Primers targeting the major capsid protein-encoding gene g23 for T4-like *myoviridae,* the major capsid protein for T7-like *podoviridae*, and the major coat protein-encoding gene for T5-like *sphirovidae* which are mostly conserved among phage families (Table 2) were utilized. PCR amplification reactions were performed in 20 μl reaction volumes consisting of 2.5 mM MgCl2, 2.5 PCR buffer, 0.2 mM each deoxynucleotide triphosphate, 1 U of Taq DNA polymerase, 1.5 μl of each primer, 3 μl of template DNA and the remaining amount of nuclease-free water. The amplification was performed in 35 cycles with an initial denaturation at 95 for 2 min followed by a denaturation step of 95 for 30 sec, primer annealing at 55 and primer extension at 72 with a final extension at 72.

**Table 2:** PCR primers used in phage identifications

Gene	Family	Sub-family	Genus	Sequence (5’→3’)	Reference
MCP	*Myoviridae*	*Tevenvirinae*	*T4-like*	T4-fw: CCC TGC TGT TCC AGA TCG ANA ARG ARG C	[35]
				T4-rev: CTG CCT GGC GTA CTG GTC DAT RWA NAC	
		*Ounavirinae*	*FO1-like*	FO1-fw: CGC CAT TGA AGA ACT GCG TRW RCA YAT GGA	
				FO1-rev: GGC ATC ATA TAG GAA TGC GCY TCR AAR TC	
MCP	*Podoviridae*	*Autographivirinae*	*T7-like*	T7-fw: GAC AAG CGG AAG GAC ATC AAN CAY ACN GAR A	
				T7-rev: CGC GTA GTT GGC GGC RTT NGG CAT NA	
McoP	*Siphoviridae*	-	*T5-like*	MCF-2F: GCGTGATGGTTGGGATGGTA	[36]
				MCF-2R: GACGCTCAATCTGACGACCA	

The amplified DNA products from phage family-specific-PCR were further analyzed using agarose gel electrophoresis to detect the presence of targeted phage DNA. The DNA samples were simultaneously checked on a 1% agarose gel along with a DNA ladder with 0.3 μL of 10 *×* loading dye, and 1*×* TAE buffer. The gel products were visualized on an ultraviolet illuminator and imaged with a gel documentation system (Bio-Rad).

### Data management and statistical analysis

The collected data were computed by using R Studio 4.1 and Excel for appropriate statistical analysis. Statistical analysis was performed to describe different variables and parameters in the study and to describe their relationship with each other as well. Descriptive statistics were used to derive percentages, and standard deviations, and to tabulate tables and graphs as well to describe the lytic bacteriophages concerning their lysis profile and their exposure to pH and temperature. Effects were reported as statistically significant as a *P* value of less than or equal to 0.05. Analyzed data were presented in pie chart, bar graphs and tables.

## Results

### Recovery of bacteriophages

**Fig. 2:**
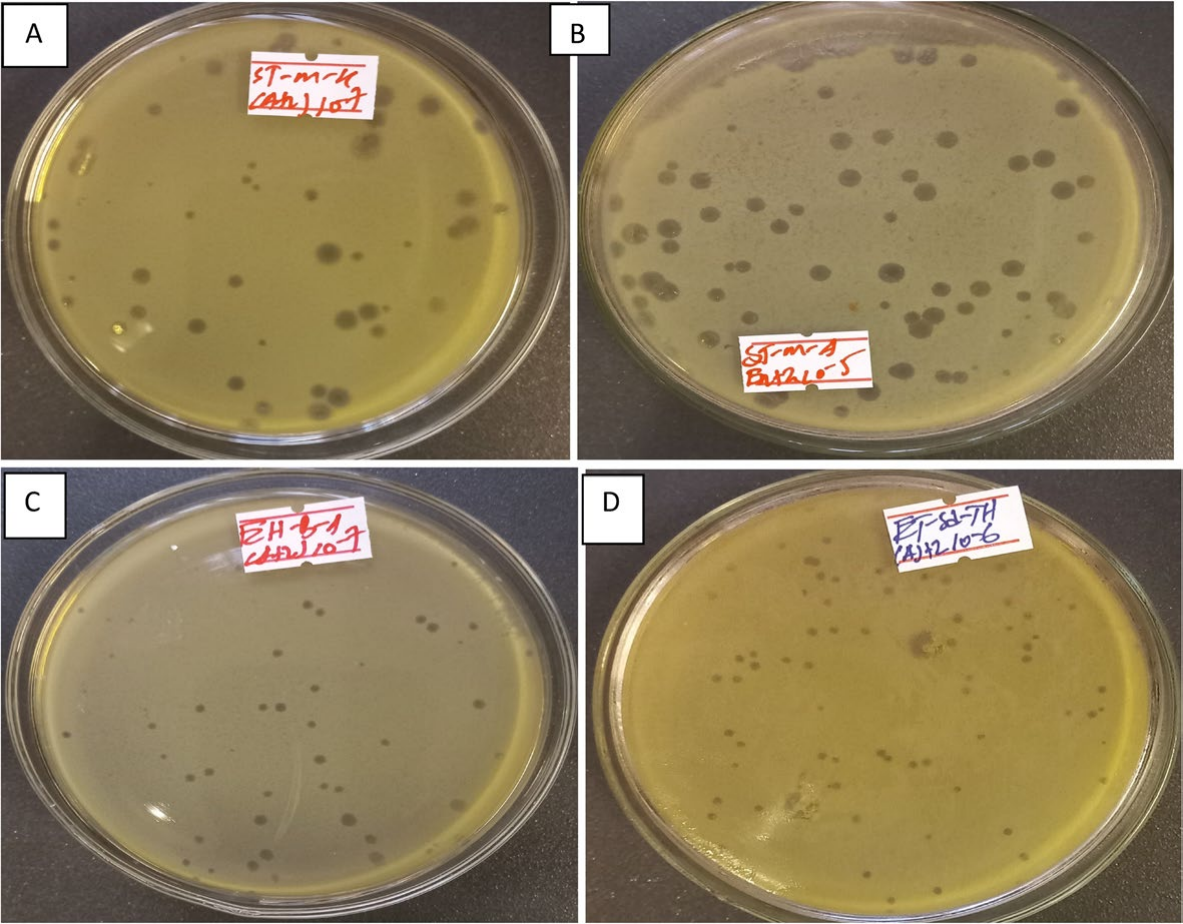
Representative bacteriophage plaque images during isolation and purification depicting different morphologies: (**A**) Clear plaque with elevated halos (**B**) Large size clear plaques (**C**) Small sized clear plaques (**D**) Small pinned clear plaques

In total, 14 samples of river water, dairy farm sewage, and hospital sewage were screened within each of the six *E. coli* strains for the presence of phages. Using 6 different *E. coli* host strains, 17 phages were isolated from 84 culture plates tested by the double agar overlay method. *E. coli* lytic phages were isolated from all the sample sites. These phages produced clear and centered plaques of different sizes (Fig. 2). All positive results were denoted as lytic bacteriophages due to the clear zone plaques on the agar. Isolated phages were named according to the *E. coli* host and sample site (e.g., phage ET-SD-TH stands for host organism ETEC and sample site sediment of Tikur Anbessa Hospital from where it was isolated). The spectrum of effective phages, their isolation site, and the hosts from which they are isolated are illustrated in Tables 3 and 4.

**Table 3:** Overall samples processed with respective phages isolated

Sample No	Sample name	Presence of lytic phages for *E. coli*	Pathogenic *E. coli* strain (s) tested
1	EA-T-K	Absent	EAEC
2	EH-T-K	Absent	EHEC
3	EI-T-K	Absent	EIEC
4	EP-T-K	Absent	EPEC
5	ET-T-K	Absent	ETEC
6	ST-T-K	Present	STEC
7	EA-M-K	Absent	EAEC
8	EH-M-K	Absent	EHEC
9	EI-M-K	Absent	EIEC
10	EP-M-K	Present	EPEC
11	ET-M-K	Absent	ETEC
12	ST-M-K	Present	STEC
13	EA-B-K	Absent	EAEC
14	EH-B-K	Absent	EHEC
15	EI-B-K	Absent	EIEC
16	EP-B-K (	Present/ 2 phages/ EP-B-K (B) & EP-B-K (E2)	EPEC
17	ET-B-K	Absent	ETEC
18	ST-B-K	Absent	STEC
19	EA-T-A	Present	EAEC
20	EH-T-A	Absent	EHEC
21	EI-T-A	Absent	EIEC
22	EP-T-A	Absent	EPEC
23	ET-T-A	Absent	ETEC
24	ST-T-A	Absent	STEC
25	EA-M-A	Present	EAEC
26	EH-M-A	Absent	EHEC
27	EI-M-A	Absent	EIEC
28	EP-M-A	Present	EPEC
29	EH-M-A	Absent	EHEC
30	ST-M-A	Present	STEC
31	EA-B-A	Absent	EAEC
32	EH-B-A (A1 & A2)	Present/2 phages/ EH-B-A (A1) & EH-B-A (A2)	EHEC
33	EI-B-A	Absent	EIEC
34	EP-B-A	Absent	EPEC
35	ET-B-A	Absent	ETEC
36	ST-B-A	Absent	STEC
37	EA-SP-GF	Absent	EAEC
38	EH-SP-GF	Absent	EHEC
39	EI-SP-GF	Present	EIEC
40	EP-SP-GF	Absent	EPEC
41	ET-SP-GF	Absent	ETEC
42	ST-SP-GF	Absent	STEC
43	EA-SD-GF	Absent	EAEC
44	EH-SD-GF	Absent	EHEC
45	EI-SD-GF	Absent	EIEC
46	EP-SD-GF	Absent	EPEC
47	ET-SD-GF	Absent	ETEC
48	ST-SD-GF	Absent	STEC
49	EA-SP-TH	Absent	EAEC
50	EH-SP-TH	Present	EHEC
51	EI-SP-TH	Absent	EIEC
52	EP-SP-TH	Absent	EPEC
53	ET-SP-TH	Absent	ETEC
54	ST-SP-TH	Absent	STEC
55	EA-SD-TH	Absent	EAEC
56	EH-SD-TH	Present	EHEC
57	EI-SD-TH	Absent	EIEC
58	EP-SD-TH	Absent	EPEC
59	ET-SD-TH	Present	ETEC
60	ST-SD-TH	Absent	STEC
61	EA-SP-SMA	Present	EAEC
62	EH-SP-SMA	Absent	EHEC
63	EI-SP-SMA	Absent	EIEC
64	EP-SP-SMA	Absent	EPEC
65	ET-SP-SMA	Absent	ETEC
66	ST-SP-SMA	Absent	STEC
67	EA-SD-SMA	Absent	EAEC
68	EH-SD-SMA	Absent	EHEC
69	EI-SD-SMA	Absent	EIEC
70	EP-SD-SMA	Absent	EPEC
71	ET-SD-SMA	Absent	ETEC
72	ST-SD-SMA	Absent	STEC
73	EA-SP-FA	Absent	EAEC
74	EH-SP-FA	Absent	EHEC
75	EI-SP-FA	Absent	EIEC
76	EP-SP-FA	Absent	EPEC
77	ET-SP-FA	Absent	ETEC
78	ST-SP-FA	Absent	STEC
79	EA-SD-FA	Present	EAEC
80	EH-SD-FA	Absent	EHEC
81	EI-SD-FA	Absent	EIEC
82	EP-SD-FA	Absent	EPEC
83	ET-SD-FA	Absent	ETEC
84	ST-SD-FA	Absent	STEC

**Table 4:** Phages recovered with respective hosts and sampling sites

No	Phage	Host	Sample site and location
1	ET-SD-TH	ETEC	Tikur Anbessa Hospital (Sediment)
2	ST-M-K	STEC	Kebena river (Medium)
3	EI-SP-GF	EIEC	Gullele farm (Superficial)
4	EA-T-A	EAEC	Akaki river (Top)
5	EP-B-K (B)	EPEC	Kebena river (Bottom)
6	EH-SD-TH	EHEC	Tikur Anbessa Hospital (Sediment)
7	EP-B-K (E2)	EPEC	Kebena river (Bottom)
8	ST-T-K	STEC	Kebena river (Top)
9	EH-SP-TH	EHEC	Tikur Anbessa Hospital (Superficial)
10	EH-B-A (A1)	EHEC	Akaki river (Bottom)
11	EH-B-A (A2)	EHEC	Akaki river (Bottom)
12	ST-M-A	STEC	Akaki river (Medium)
13	EA-M-A	EAEC	Akaki river (Medium)
14	EP-M-K	EPEC	Kebena river (Medium)
15	EP-M-A	EPEC	Akaki river (Medium)
16	EA-SP-SMA	EAEC	Shiromed area farm (Superficial)
17	EA-SD-FA	EAEC	Ferensay area farm (Sediment)

**Fig. 3:**
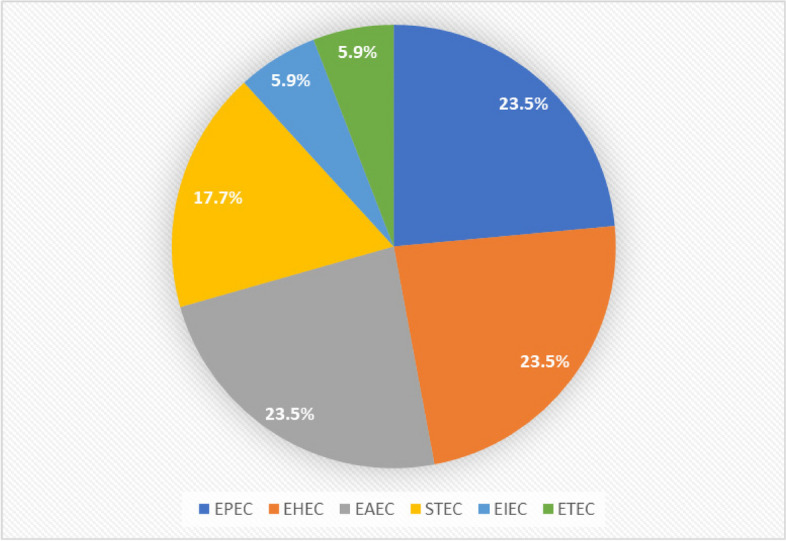
Frequency of lytic phages isolated against the six pathogenic *E. coli* strains

**Fig. 4:**
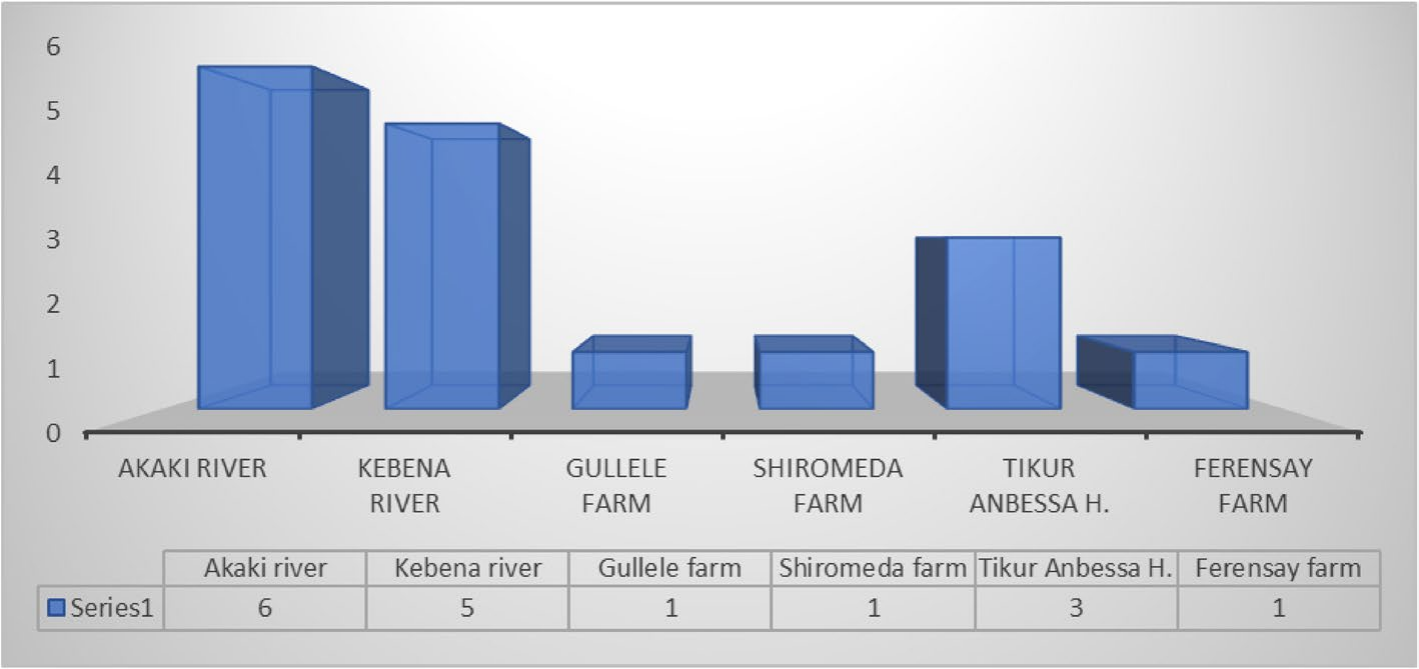
Frequency of lytic phages recovered from the sample sites

The number of phage strains recovered was 3 (17.7%) against STEC, 1 (5.9%) against both ETEC and EIEC, and 4 (23.5%) against EPEC, EHEC, and EAEC (Fig. 3). The recovery of phages was slightly higher in river water samples than in dairy farm and hospital wastes, and the concentration of phage was greater in the bottom and middle layers than in the superficial and/or top layers of the collection tank (Fig. 4; Table 3).

### Bacteriophage titer determination

Bacteriophage titers were observed between 2.8 × 10^7^ and 3.12 × 10^10^ PFU mL − 1 (Table 5). The highest titer of bacteriophage was isolated from the EP-M-K bacteriophage with a titer of 3.12 × 10^10^ PFU mL − 1. The titer of phages from the medium and bottom of the river and sediment surface of the dairy farm, as well as hospital waste, was highly concentrated compared to the top surface of the river and superficial surface of farm and hospital fluid waste.

**Table 5:** Phage titers and corresponding plaque morphology

No	Phages	Titer (PFU/ml)	Plaque morphology
1	EP-M-K	3.12 × 10^10^	Clear plaque with small halos
2	EP-B-K (B)	6.0 × 10^9^	Haloed clear plaques
3	EP-B-K (EN2)	1.7 × 10^9^	Clear plaque with halos
4	EP-M-A	2 × 10^10^	Clear plaque with diffused surrounding
5	EA-SP-SMA	3.0 × 10^10^	Elevated-type haloed plaque
6	EA-T-A	1.4 × 10^9^	Haloed clear plaques
7	EA-SD-FA	3.1 × 10^10^	Clear plaque with halo surrounded
8	EA-M-A	12.0 × 10^9^	Small clear plaques
9	EH-B-A (A1)	5.1 × 10^7^	Small-sized clear plaques
10	EH-B-A (A2)	12.0 × 10^9^	Clear plaque with small pin headed
11	EH-SD-TH	7.5 × 10^7^	Medium size clear plaques
12	EH-SP-TH	9.8 × 10^9^	Large size clear plaques
13	EI-SP-GF	9.0 × 10^7^	Small size clear plaques
14	ET-SD-TH	2.8 × 10^7^	Small clear plaques
15	ST-T-K	17.5 × 10^8^	Large-sized diffused plaques
16	ST-M-A	8.4 × 10^9^	Large-sized clear plaques
17	ST-M-K	16.5 × 10^8^	Clear-centered plaque with halos

### Morphology of phages analyzed by SEM

**Fig. 5:**
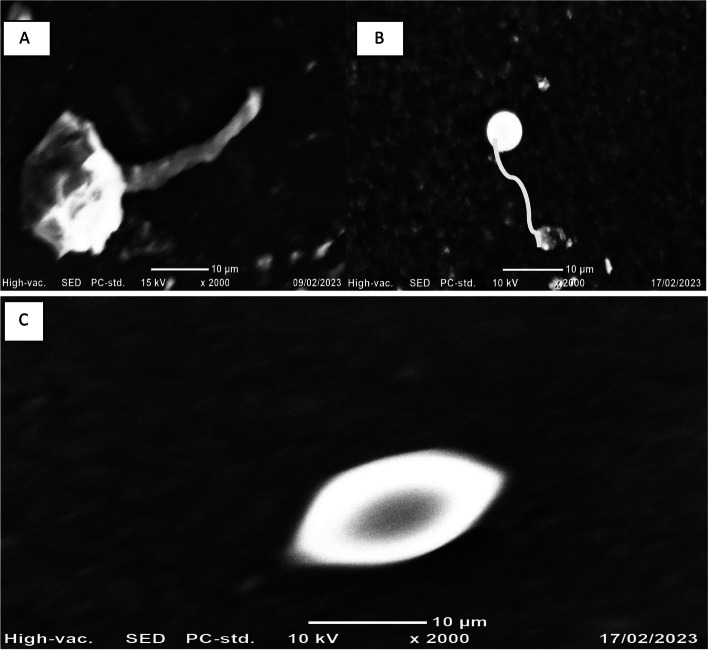
Scanning electron microscopy representative images of phage families: (**A**) *Myoviridae* like phage (**B**) *Siphoviridae* like phage (**C**) *Podoviridae* like phage morphology

Seventeen phage isolates were subjected to SEM analysis to determine their morphotype. Scanning electron micrograph images of the phages and structural dimensions are shown in Fig. 5. Phage isolates were classified as per the International Committee on Taxonomy of Virus (ICTV) classification based on the three-dimensional structure observed. Only one phage isolate (EP-M-K) was identified clearly; three related phages (EA-SD-FA, ST-M-A & EH-SP-TH) were also identified by using SEM imaging of the isolates. The identified phage isolates were in the order *Caudovirales* of three families; *Myoviridae*, *Siphoviridae, and Podoviridae*. Among the 4 phage isolates, 2 were classified as *Myoviridae*-like phages while 2 were classified as *Siphoviridae*-like and *Podoviridae*-like phages. *Myoviridae* phages have relatively large icosahedral heads and a long, thick, complex, contractile tail, consisting of a central tube surrounded by a contractile sheath and ancillary structures. *Siphoviridae* consists of a long noncontractile thin flexible tail with short, kinked, terminal fibers (Fig. 5B). *Podoviridae* phages have nonenveloped icosahedral heads with short, noncontractile tails (Fig. 5C).

### Description of bacteriophage isolates

#### Host range test

**Fig. 6:**
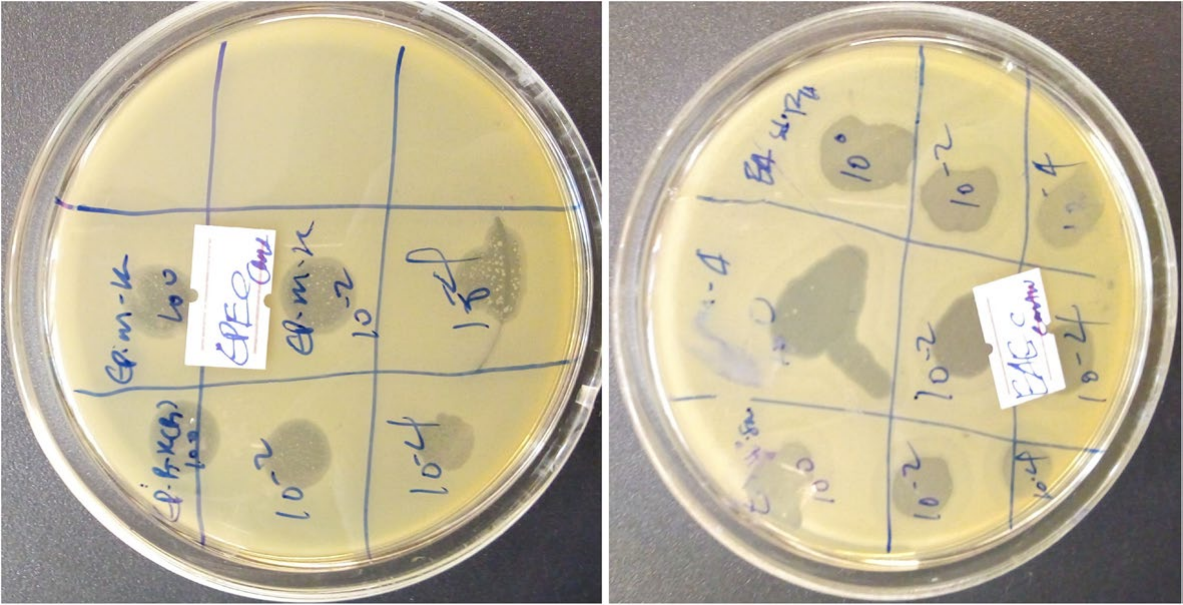
Analysis of the separated phages’ host range representative image: The spot lysed by different phages that depict a clear lysis zone on a TSA agar plate

Phages were selected based on the size and clarity of plaques they produced for screening their host range to determine the lytic profile (Fig. 6). Thus, the infectivity of 17 phages was analyzed against 3 other pathogenic bacterial species including *Salmonella Typhimurium*, *Shigella flexineri, Klebsiella pneumoniae,* and other *E. coli* isolates apart from host strains*.* The host range of these phages was investigated by the spot method, which revealed that most of the phages were able to lyse pathogenic strains.

The results in Table 6 show that 11 (65%) of the phages were effective against available bacterial species since clear plaques were formed when phages were spotted on the bacterial lawn. However, 6(35%) of the phages demonstrated no lysis and were not able to produce areas of clear zones on a bacterial lawn. Among 17 phages, EP-M-A was the most effective phage with 62.5% lytic ability killing 5 different bacterial strains, followed by phage EI-SP-GF which lysed 4 different strains. The intensity of EP-M-A phage was effective in *Klebsiella pneumoniae, Salmonella Typhimurium, Shigella flexineri, EAEC, and STEC* whereas EI-SP-TH was effective in *Salmonella Typhimurium, Shigella flexineri, EHC and STEC.* Phages EP-B-K (B), EH-SD-TH, ET-SD-TH, EH-B-A (A1), and EH-B-A (A2) were each able to lyse three bacterial hosts while phages ST-M-A and EA-M-A were each able to lyse two *E. coli* strains. Phages EP-M-K and ST-M-K lysed only one bacterial host when compared to other phages. Contrary to the low levels of virulence displayed by these phages, seven phages (EP-M-A, EI-SP-GF, EP-B-K (E2), EH-SD-TH, ET-SD-TH, EH-B-A (A1), and EH-B-A (A2)) demonstrated the broader spectrum of activity since they could be infected between 3 and 5 bacterial host strains. These seven superphages were selected for further characterization.

**Table 6:** Host range of phages isolated on *E. coli* hosts

Phages	Test organisms of bacterial species and other *E. coli* strains								
	*Sal. typhimurium*	*Sh. flexinari*	*K. pneumoniae*	*EPEC*	*ETEC*	*EHEC*	*EIEC*	*EAEC*	*STEC*
EP-M-A	+	+	+	H	–	–	–	+	+
EP-M-K	–	–	–	H	–	–	–	–	+
EP-B-K (B)	–	–	–	H	–	–	–	–	–
EP-B-K (E2)	+	–	+	H	–	–	–	–	+
EH-SD-TH	+	–	–	–	+	H	–	–	+
ET-SD-TH	+	+	–	–	H	+	–	–	–
EI-SP-GF	+	+	–	–	–	+	H	–	+
EH-B-A (A1)	+	+	–	–	–	H	–	–	+
EH-SP-TH	–	–	–	–	–	H	–	–	–
EH-B-A (A2)	+	+	–	–	–	H	–	–	+
ST-T-K	–	–	–	–	–	–	–	–	H
ST-M-A	–	–	–	+	–	–	–	+	H
EA-SP-SMA	–	–	–	–	–	–	–	H	–
EA-SD-FA	–	–	–	–	–	–	–	H	–
ST-M-K	–	–	–	+	–	–	–	–	H
EA-M-A	–	–	–	+	–	–	–	H	+
EA-T-A	–	–	–	–	–	–	–	H	–

+ lysis, − no plaques, H host isolated

#### Efficiency of plating (EOP) in seven phages

**Fig. 7:**
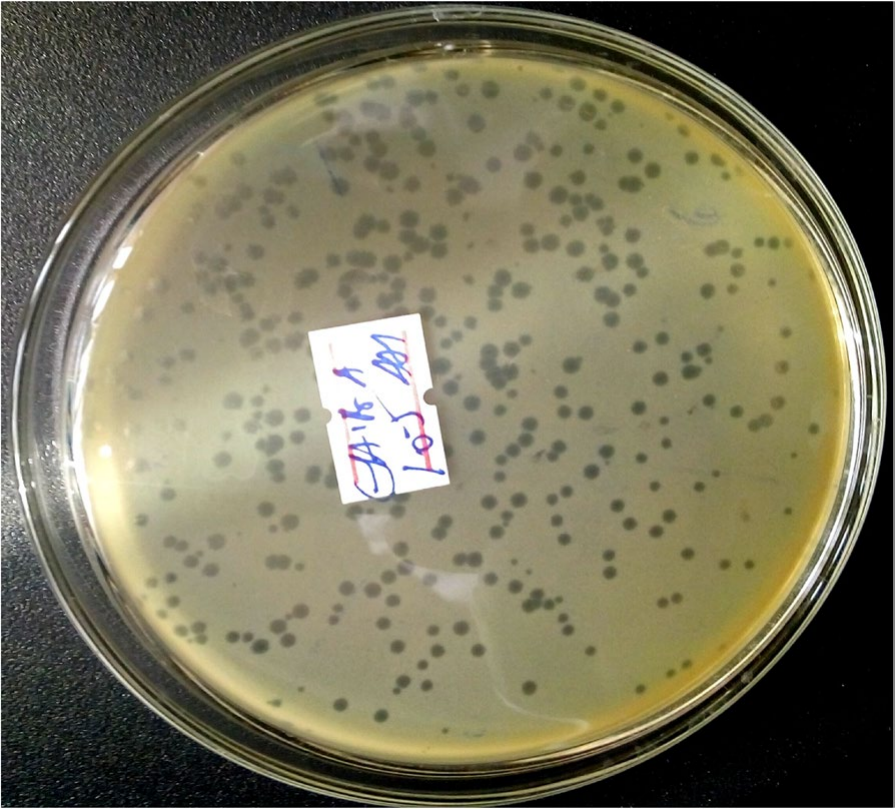
Representative image of phage plaques in the EOP reference host

The EOP analysis was performed on four bacterial strains including *Klebsiella pneumoniae, Salmonella Typhimurium, Shigella flexineri,* and *STEC,* that were susceptible to phages in the spot test. The EOP was calculated as the ratio between the average number of plaques of target host bacteria (PFUs) and the average number of plaques of reference host bacteria (PFUs). The EOP was classified as high (EOP ≥ 0.5), moderate (EOP > 0.1 < 0.5), and low ((EOP ≤ 0.1) based on the reproducible infection of the target bacteria. Although spot test results revealed clear plaques on reference hosts (Fig. 7), EOP results exhibited various lytic patterns of the phages. Even though EOP analysis revealed high (EOP ≥ 0.5) productive infection on reference bacterial hosts, moderate and low infections were observed (Table 7). Four phages (EH-SD-TH, EI-SP-GF, EP-M-A, EH-B-A (A1)) revealed high EOP values (0.7–1.4) on reference hosts. On the other hand, EOP analysis exhibited moderate and low productive infections on the hosts’ EOP values ranging from 0.1 to 0.5 and less than 0.1 respectively.

**Table 7:** Efficiency of plating for spot test effective phages

Phages	Reference host	Titer in isolate host	Titer in EOP test sp.	EOP
EP-M-A	*Shigella flexineri*	2 × 10^10^	2.8 × 10^9^	0.7
ET-SD-TH	*Salmonella typi.*	2.8 × 10^7^	1.1 × 10^8^	0.2
EH-SD-TH	*STEC*	7.5 × 10^7^	5.2 × 10^7^	1.4
EH-B-A (A1)	*STEC*	5.1 × 10^7^	4.4 × 10^8^	1.2
EI-SP-GF	*Shigella flexineri*	9.0 × 10^7^	3.2 × 108	1.38
EH-B-A (A2)	*Shigella flexineri*	12.0 × 10^9^	3.0 × 10^10^	0.4
EP-B-K (E2)	*Klebsiella pneumoniae*	1.7 × 10^9^	1.8 × 10^9^	0.09

#### Optimal multiplicity of infection (MOI)

The optimal MOI was determined as the ratio of the number of phages to that of host bacteria present in a defined space that is best for phage proliferation to obtain maximum titers. *E. coli* cultures of the exponential growth phase were infected with different amounts of phages with a set of serial dilutions at 10 (10/1), 1 (1/1), 0.1 (1/10), 0.01(1/100), 0.001(1/1000), and 0.0001(1/10,000). The phage titers were measured after incubation for 2 h. The results indicated that the optimal MOIs of phage isolates EP-M-A, EI-SP-GF, EP-B-K (B), EH-SD-TH, ET-SD-TH, EH-B-A (A1), and EH-B-A (A2) were 1, 0.1, 0.1, 0.01, 0.1, 0.01 and 1, respectively, which gave the highest production of phage progeny (Table 8). Phage EP-M-A had the highest MOI (1) which was also comparable to the host range test result, but EI-SP-GF had a broad host range with a slight difference in MOI. In general, phage with a broader host range had the highest MOI and vice versa.

**Table 8:** The multiplicity of infection in phages isolates

*E. coli* strain (CFU/500 ul)		MOI	Phage isolates titer (PFU/ml) after infection						
			EP-M-A	EP-B-K	EH-SD-TH	ET-SD-TH	EH-B-A (A1)	EH-B-A, (A2)	EI-SP-GF
EPEC	1.8 x 10^10^	10	1.3 x 10^8^	2.7 x 10^8^	5.7 x 10^7^	6.1 x 10^5^	2.7 x 10^7^	8.4 x 10^8^	9.1 x 10^5^
ETEC	3.4 x 10^8^	1	**2 x 10**^**10**^	3.9 x 10^7^	3.9 x 10^7^	1.6 x 10^7^	3.3 x 10^7^	**12.0 x 10**^**9**^	8.6 x 10^7^
EHEC	8.4 x 10^8^	0.1	1.2 x 10^9^	**1.7 x 10**^**9**^	1.5 x 10^6^	**2.8 x 10**^**7**^	2.4 x 10^6^	2.6 x 10^8^	**9.0 x 10**^**7**^
EIEC	5.2 x 10^7^	0.01	3.7 x 10^8^	1.5 x 10^8^	**7.5 x 10**^**7**^	2.7 x 10^7^	**5.1x 10**^**7**^	7.3 x 10^9^	7.7 x 10^7^
EAEC	3.4 x 10^5^	0.001	7.2 x 10^5^	1.7 x 10^8^	8.1 x 10^5^	3.9 x 10^6^	7.1 x 10^5^	5.4 x 10^8^	8.3 x 10^7^
STEC	1.2 x 10^6^	0.0001	6.7 x 10^7^	3.5 x 10^6^	4.3 x 10^7^	1.4 x 10^6^	4.6 x 10^7^	6.2 x 10^7^	9.4 x 10^6^

#### One-step growth curve of phages

**Fig 8:**
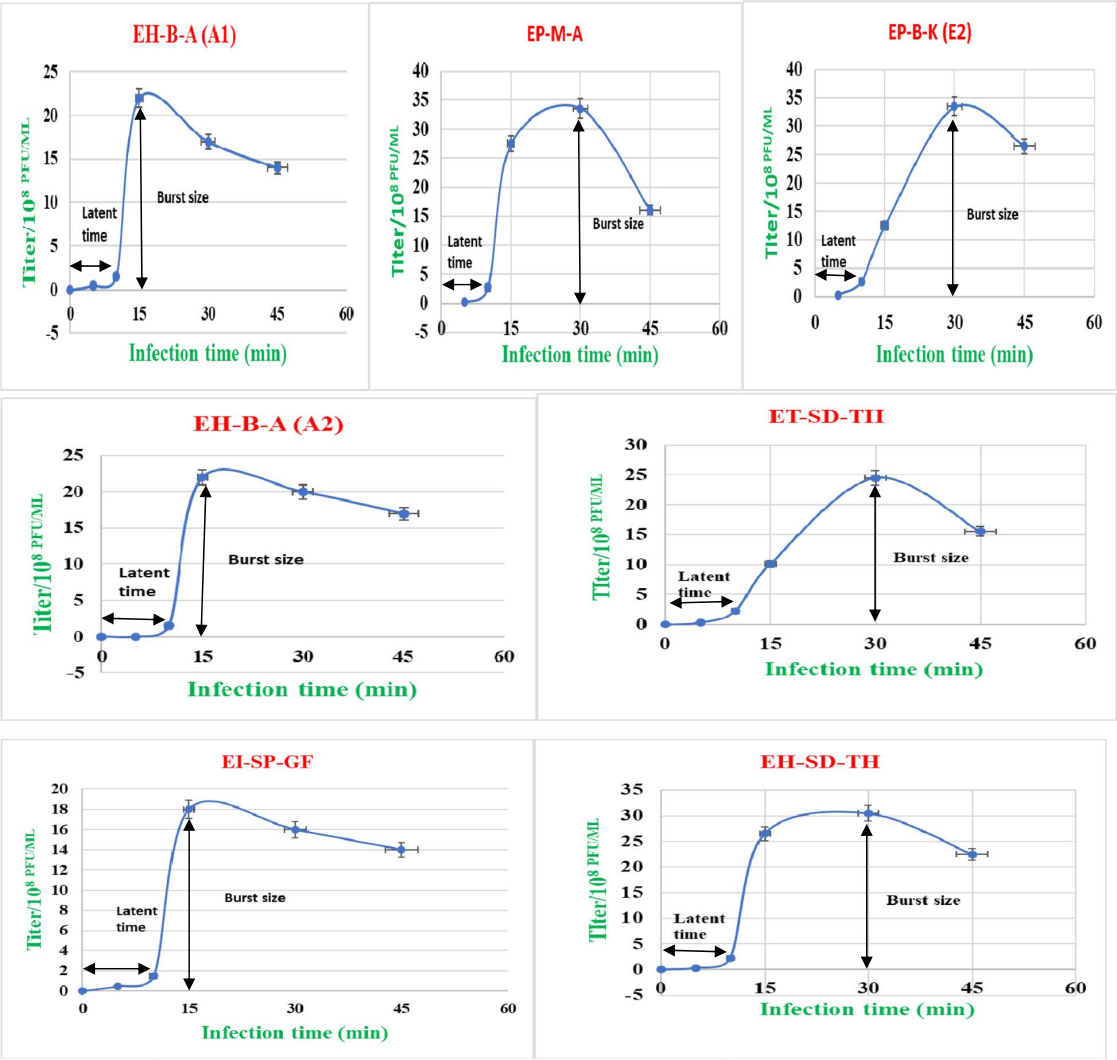
One-step growth curves in seven potent phages: The double-layer agar method was used to calculate the phage titers. Latent periods and burst sizes were estimated from the graph in which an arrow line indicated for each phage. **A** EH-B-A (A1) phage growth curve; (**B**) EP-M-A growth curve; (**C**) EP-B-K (E2); (**D**) EH-B-A (A2); (**E**) ET-SD-TH; (**F**) EI-SP-GF; (**G**) EH-SD-TH phage growth curve. The average of two separate trials is shown by each data point. Standard deviations between the duplicate samples are displayed by error bars

The latent duration and relative burst size per infected bacterial cell were determined using a one-step growth curve analysis for the seven phage isolates using the optimal MOI. Triphasic curves were created using the data generated after analysis (Fig. 8). All phages had a latent duration between 10 and 15 min followed by a burst period between 15 and 30 min. The relative burst size, defined as the mean phage titer value at the plateau phase divided by that of the latent phase, was approximately between 87 and 364 virions per infected cell. Phages EH-B-.

A (A1) and EP-B-K (E2) exhibited the largest burst sizes (364 and 268 PFUs, respectively) per infected cell; whereas phage ET-SD-TH had the smallest burst size (87 PFUs). The mean burst sizes of phages EH-B-A (A2), EI-SP-GF, EH-SD-TH, and EP-M-A (were 200, 193, 105, and 129 PFUs, respectively).

#### Temperature and pH stability

Plaque-forming units (PFUs) were used to measure changes in survival to evaluate the pH and thermal stabilities of phages. After incubating the phage at temperatures ranging from 25 to 90 °C for 6 h, the thermal stability of the phage isolates was assessed using a phage titration experiment with 37 °C as the isolation temperature point. In the case of the pH test, phages were also incubated at different pH values of phage buffer ranging from 3 to 11 at 37 °C for 6 h with the pH of the isolation, with pH 7.5 as medium.

**Fig. 9:**
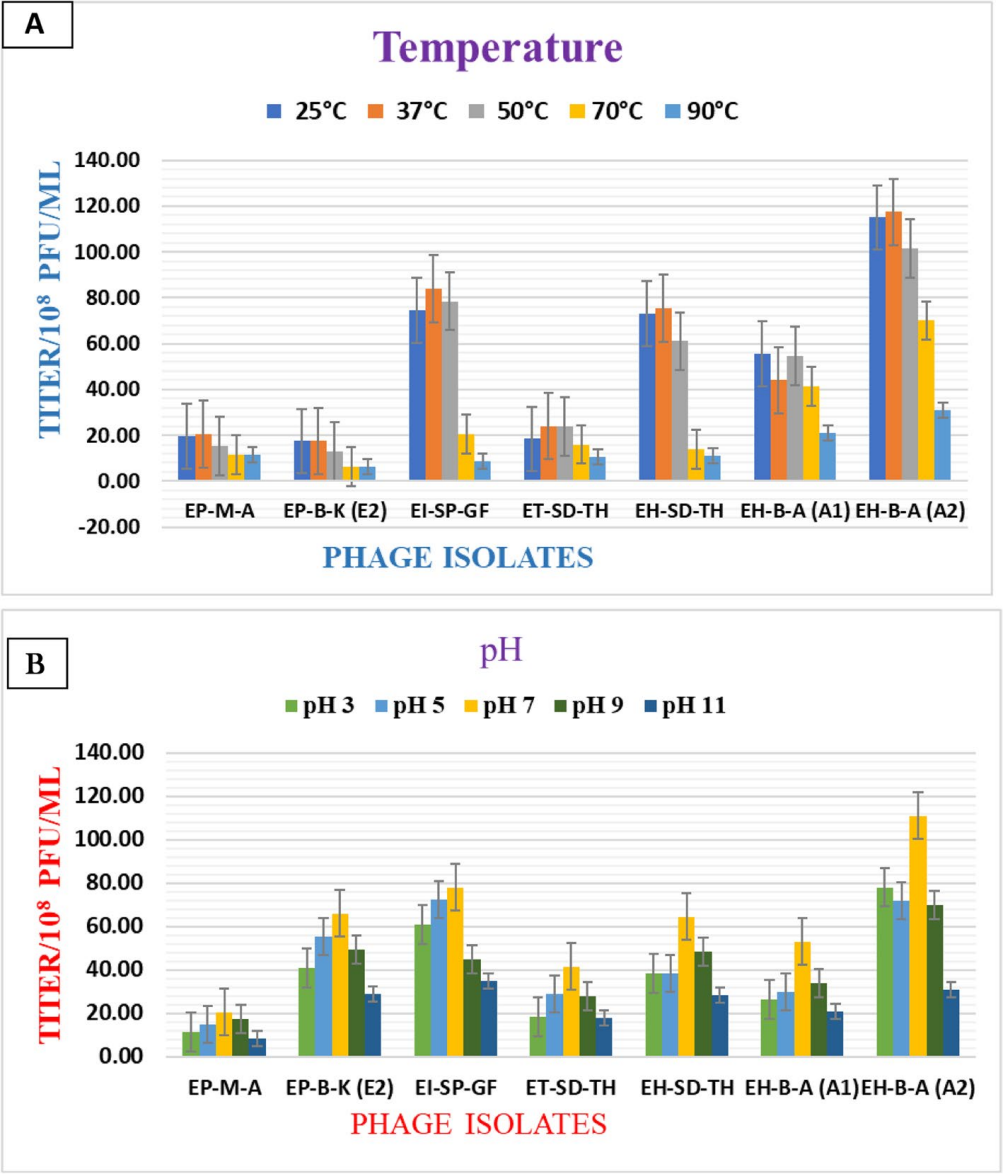
Stability of *E. coli* lytic phages at different temperature and pH levels: (A) Phages were incubated at various temperatures between 25 and 90 °C. (B) pH sensitivity test at different pH ranges between 3 and 11. The average of two separate experiments was used to draw the graph. Vertical lines in the graph represent standard deviations

According to the results of thermal stability tests, all phages were stable at temperatures between 25 °C and 70 °C and did not become less viable after being incubated for 6 h at the appropriate temperatures except EP-B-K (E2) and EH-SD-TH, which showed a high loss in titer at 70 °C. The infectivity of the phage was entirely observed to decrease after incubation at 90 °C for 6 h in all isolates. When the temperature increased, the viability of the phage significantly decreased. All phages had optimum or good infectivity at 37 °C (Fig. 9A).

The results of pH stability tests showed that phage viability was largely unaltered following incubation in buffer at pH values ranging from 5 to 9 with a slight loss at pH 9 particularly for EI-SP-GF and ET-SD-TH, while decreases in titer were roughly recorded at pH 3 and pH 11 in all phage isolates (Fig. 9B). EH-B-A (A2) was the most stable phage at pH 3. After 6 h of incubation, all phages survived well at pH 7 and pH 9 with no appreciable drop in titer. A pH of 7 and a temperature of 37 °C were the best optimum conditions for phages. These findings demonstrate the wide temperature and pH tolerance of the phage isolate.

### Molecular characterization of phages

#### DNA extraction and analysis

DNA was successfully extracted from 17 phages that were lytic against diarrheagenic *E. coli strains*. The range of the DNA concentrations was 58.6–1241.9 ng/L. The amount of DNA that was extracted and the initial phage concentration were not linearly correlated. For example, the highest titer of bacteriophage was isolated from EP-M-K phage with a titer of 3.12 × 10^10^ PFU mL – 1; whereas, the highest DNA concentration was extracted from EP-B-K (E2) which was 1241.9 ng/L.

The measurement of DNA preparations revealed that 12 out of 17 DNA samples were high quality and others were relatively acceptable. An intact band indicates that the DNA has not been damaged or tainted with host RNA and DNA. The result of a possible contaminant check revealed that phage samples treated with DNase and RNase had some clear gel images whereas those samples that were not treated with DNase and RNase showed smear or indistinct gel images.

#### PCR based identification of phages

**Fig. 10:**
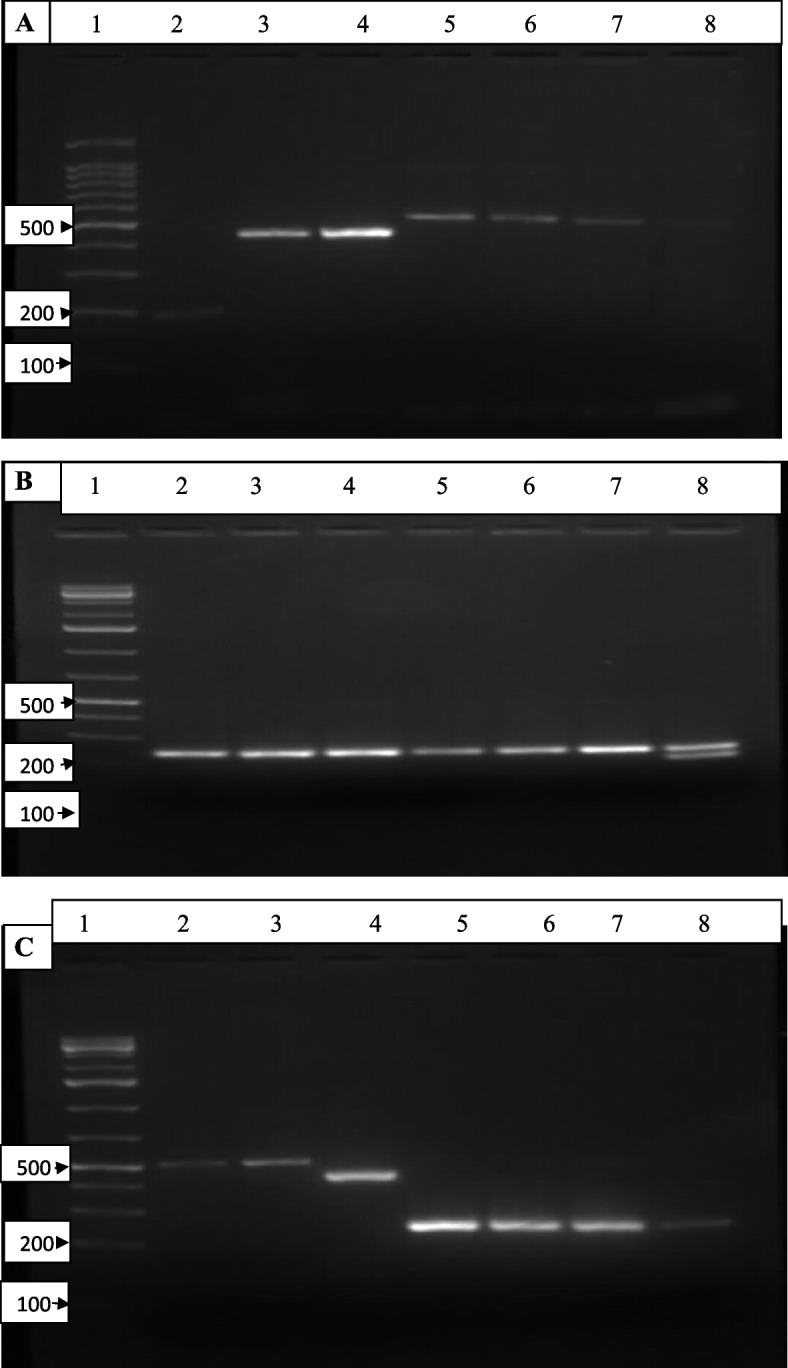
Gel images of PCR identified phage isolates: (**A**) Lane-1, 1 kb plus ladder; Lane-2, T5; Lane-3 & 4, T7 and Lane-5 to 8, FO1 phages with 200 bp, 461 bp, and 519 bp respectively (**B**) Lane-1, 1 kb plus ladder; Lane-2 to 8 all phages were T4 phages with 240 bp size (**C**) Seven potent phages selected in separate gel; Lane-1, 1 kb plus ladder; Lane − 2 & 3, 519 bp sized FO1 phages of EP-M-A & EP-B-K (E2), Lane-4, 461 bp sized T7 phage EI-SP-GF and Lane- 5 to 8, 240 bp sized phages of EH-SD-TH, EH-B-A (A1), EH-B-A (A2), ET-SD-TH

Out of 17 phage isolates, 15 were identified by PCR targeting the major capsid protein of *myoviridae* and *podoviridae* phages as well as the major coat protein of *siphoviridae* phages. The *Siphoviridae* and *Podoviridae* families were detected with amplicon sizes of 200 bp and 461 bp, respectively. Moreover, *Myoviridae* was detected with different amplicon sizes of 240 bp or the T4 virus and 519 bp or the Felixo 1 virus (Fig. 10A & B). Of 15 identified phage isolates two (EI-SP-GF and EA-T-A) were in the family of *Podoviridae* and the other two (EA-SP-SMA and ST-M-A) were in the family of *Siphoviridae*. The remaining phages were in the family of *Myoviridae* with four phages (EP-M-K, EP-M-A, EP-B-K (B), and EP-B-K (E2)) identified as Felixo 1 virus and seven phages (EH-SD-TH, ET-SD-TH, EH-B-A (A1), EH-B-A (A2), EA-M-A, EA-SD-FA, and ST-M-K) identified as T4 virus. Similarly, among seven potent phage isolates six were *myoviridae* phages, four of which were T4 viruses, and 2 of which were FO1 phage viruses (Fig. 10C). PCR unidentified phage isolates were ST-T-K and EH-SP-TH.

According to the PCR results, approximately 73% of phage isolates were *myoviridae*-like phages with 47% being T4 phages and 26% of phages being FO1. *Podoviridae*-like and *Siphoviridae*-like phages were 27% with 13.5% each identified using PCR (Table 9). Therefore, *Myoviridae* phages were the most dominant coliphages isolated from different environmental samples. The EI-SP-GF *Podoviridae* phage was the only phage isolated among seven potent phages.

**Table 9:** PCR-identified phage isolates with their family and genus

Phages	Family	Genus	Amplicon size
EP-M-A	*Myoviridae*	Felixo 1 virus	519 bp
EP-B-K/B	*Myoviridae*	Felixo 1 virus	519 bp
EP-B-K/EN2	*Myoviridae*	Felixo 1 virus	519 bp
ET-SD-TH	*Myoviridae*	T4 virus	240 bp
EH-SD-TH	*Myoviridae*	T4 virus	240 bp
EH-B-A/A1	*Myoviridae*	T4 virus	240 bp
EH-B-A/A2	*Myoviridae*	T4 virus	240 bp
ST-M-A	*Siphoviridae*	T5 virus	200 bp
ST-M-K	*Myoviridae*	T4 virus	240 bp
EI-SP-GF	*Podoviridae*	T7 virus	461 bp
EA-T-A	*Podoviridae*	T7 virus	461 bp
EA-SD-FA	*Myoviridae*	T4 virus	240 bp
EA-SP-SM	*Siphoviridae*	T5 virus	200 bp
EA-M-A	*Myoviridae*	T4 virus	240 bp
EP-M-K	*Myoviridae*	Felixo 1 virus	519 bp

## Discussion

Pathogenic *E. coli* is known to cause diarrhea and related diseases in both animals and humans. The emergence of antibiotic resistance has led to renewed interest in exploring lytic bacteriophages as a natural and eco-friendly biocontrol strategy. These phages have the ability to lyse multidrug-resistant pathogens, making them an effective tool in eliminating pathogenic bacteria [37]. Consequently, there is a growing interest in searching for and utilizing lytic bacteriophages for the treatment of these harmful pathogens [38]. This study focused on the isolation and characterization of lytic phages against multidrug-resistant *E. coli* strains from various sources and evaluated their therapeutic potential. Bacteriophages are widely distributed in the environment, including rivers, soil, sewage, animal feces, water ponds, and seawater where their hosts reside. In Ethiopia, river water and sewage are heavily contaminated with fecal and waste matter, resulting in a high diversity of enteric organisms. This study aimed to isolate phages from relevant sources, such as river water, dairy sewage, and hospital liquid waste samples. Other studies have also successfully isolated phages from freshwater ponds, animal waste, and soil. For instance, Shukla et al. (2014) isolated phages from animal waste collected from different livestock farms, while Alonso et al. (2002) isolated 26 phages from water samples of the Alboran Sea, Western Mediterranean. Leta et al. (2017) and Betemaryam. (2020) also successfully isolated lytic phages from sewage water collected in Jimma town, Jimma, and National Veterinary Institute, Bishoftu respectively in Ethiopia against *E. coli* [39–41]. These findings suggest that phages can be isolated from a wide range of sources.

Before successfully isolating suitable lytic bacteriophages as antimicrobial agents, it is necessary to isolate, identify, and fully characterize the bacterial host [42]. In this particular investigation, multidrug-resistant pathogenic *E. coli* strains were determined to be resistant to most available antibiotics and available as glycerol stocks at the Institute of Biotechnology, Addis Ababa University. As a potential alternative and effective treatment for diseases caused by these resistant *E. coli* strains, phages were employed. Thus, 17 lytic phages were successfully isolated from various sources including river water, hospital fluid waste, and dairy farm sewage. Multidrug-resistant diarrheagenic *E. coli* strains were used as hosts. The selected phages were those that formed clear zones of lysis or plaques on the bacterial lawn, indicating their virulence and suitability for biocontrol applications. Different sizes of clear and discrete plaques were observed, ranging from small to large with phage titers ranging from 2.8 × 10^7^ to 3.12 × 10^10^ PFU/ml. Interestingly, 65% of phage isolates produced large and clear plaques on their preferred hosts, similar to *E. coli* O157, *Listeria*, *Pseudomonas,* and *Salmonella*-specific phages [30, 43]. All phage isolates were unique with respect to their host *E. coli* strain, but from some sample sites, two or more phages were detected from different partitions, such as the middle and top surfaces of the Kebena River. The main reason for this is that phages have similar receptor binding with different *E. coli* strains [44]. To determine their therapeutic value, phage properties such as host range, stability, growth kinetics, and viral yield must be characterized.

The selection of phages for the biocontrol of AMR pathogens is primarily based on the host range, which is considered a crucial factor [35]. The lytic spectra of a phage, which is a significant biological characteristic, refers to the range of bacteria genera, species, and strains that a phage can kill. In biocontrol applications, it is essential to select virulent phage candidates with broad lytic spectra instead of temperate and narrow lytic ones [45] because temperate phages have the ability to transfer virulence or antibiotic resistance genes [46], and narrow lytic ones cannot cover many bacterial strains.

The diversity among phages was evident in this study as their lytic profiles varied when different host strains of *E. coli* and other gram-negative Enterobacteria were used. Seventeen phages underwent spot tests to determine their host range, based on lytic profiles, plaque clarity, and phage size. The phages were capable of infecting different *E. coli* strains and gram-negative Enterobacteria. Interestingly, seven phages (7/17) exhibited clear plaques on different hosts, suggesting that they were polyvalent and had broader host ranges. EOP analysis revealed that four (4/7) phages had high efficiency (EOP ≥ 0.5) on the reference host strains. Although all seven phages formed clear plaques on *E. coli* hosts and gram-negative Enterobacteria species during the spot test, three phages exhibited medium to low EOP (< 0.5) on the reference host, indicating that they were highly specific to the isolation host strain. The infectivity variation might be due to non specific binding receptors on the host cell wall or the presence of phage-resistant strains. Host specificity is considered a desirable characteristic for selecting therapeutic phage applications, particularly in live animals, to ensure that they have little or no impact on the beneficial gut microflora [31].

The phages were further characterized by means of a one-step growth experiment to determine their infection characteristics, including latent period and burst size. A one-step growth curve was a graphical representation of the various stages of a phage infection cycle within a host cell population over time. This study provides insights into the dynamics of viral replication and the progression of infection. Typically, a one-step growth curve consists of a series of data points plotted against time. These parameters are important in assessing a phage’s efficacy in infecting and developing within a specific host.

The average latent period of the phages was found to be between 10 and 15 min, with burst sizes ranging from 87 to 364 particles per cell. The observed burst sizes were considered large compared to other *E. coli* phages, where average burst sizes as small as 33 and 51 pfu/cell have been reported [47]. However, a burst size of 9000 pfu/cell has been reported for the *Podovirus* phage phiAxp-3 [48]. Differences in the latent period and burst size of phages can be attributed to host cells, growth medium, pH, and temperature of incubation [25]. The short latent period and large burst size of the seven potent phages suggest that they have a competitive advantage over other phages, as they can produce enough virions to lyse host bacteria in a short amount of time. Therefore, these phages possess favorable characteristics that make them attractive candidates for a biocontrol treatment program.

Apart from biological properties, newly isolated phages should be evaluated for their stability and persistence in different environmental conditions to confirm their biocontrol potential [45]. Therefore, this study aimed to investigate the response of seven potent phages to physicochemical stress factors that might be encountered during phage production or biocontrol application. The seven phages showed similar behavior and stability at different incubation temperatures and pH values. They were stable between 25 °C and 70 °C for 6 h, but temperatures above 70 °C resulted in a reduction in titers, possibly due to the effect of high temperatures on phage proteins [49]. Similar observations have been reported in studies investigating the effect of temperature on phage stability [28]. The thermal stability of the phages to high temperatures (50 °C–70 °C) in this study suggests that they could be suitable for biocontrol applications against pathogenic *E. coli* strains.

The infectivity of *E. coli* phages is affected by acidic environments, which can lead to denaturation of phage proteins and subsequent loss of viability. Previous studies have shown that most tailed phages remain stable at pH levels between 5.0 and 9.0 [32], which is similar to the results of this study. While all of the isolated phages showed high resistance to acidic and alkaline conditions (pH 5.0 to pH 9.0) after 6 h of exposure, some phages experienced a loss of titer at pH levels of 3.0 and 9.0. However, phages EP-M-A and EP-B-K exhibited resistance to higher alkaline environments (pH 9.0), similar to previous findings on the preference of *Podoviruses* for alkaline conditions and their sensitivity to acidic conditions [50]. This alkaline stability could expand the potential applications of these phages.

SEM analysis is a rapid and simple method of characterizing phages, aiding in the identification of novel phages and attribution to families [36]. SEM observation indicated that 4 out of 17 phages, isolated from different samples, belonged to the order *Caudovirales*, with 2 of the isolates being part of the *Myoviridae* family. This family is one of the three main families of *Caudovirales,* also known as tailed phages. Although previous research has shown that tailed phages (*Caudovirales*) represent the most diverse, numerous, and widespread of all bacterial viruses, the families *Siphoviridae* and *Myoviridae* are the most prevalent, accounting for 86% of the order, while the *Podoviridae* family is the least represented, accounting for approximately 14% [49]. Similarly, studies on the morphology of environmental *E. coli* O157:H7 bacteriophages have shown the dominance of the *Myoviridae* and *Siphoviridae* families [47]. The remaining 13 phages were not identified by SEM analysis due to various factors such as phage degradation during lyophilization processes, SEM imaging errors, and sample handling. It provides detailed structural information about the phage, such as its size, shape, and surface characteristics. SEM can quickly identify the presence of phages in a sample and provide visual evidence of their morphological features. However, SEM alone cannot provide information about the specific genetic makeup or identity of the phage [51]. The PCR assays presented in this report offer the benefit of detecting the presence of *E. coli* phages and their family and genus directly from phage lysate samples in a single reaction. In this investigation, the major capsid protein and major coat protein were utilized as molecular markers to promptly classify new phages into a certain group, thereby providing a preliminary identification of their family and genus which is similar to Hopkins et al. (2014) and Born et al. (2019) who targeted the MCP gene for the PCR identification of their phage isolates [52]. The sequences of virulent phages and their genus-specificity were the basis for the selection of all primers used in this investigation. The specificity of each primer set is at a specific taxonomic level based on the current taxonomy of these phages [53].

Fifteen out of 17 phage isolates were identified by PCR amplification using specific primers. Among these 15 phages, 11 phages were in the *Myoviridae* family. This suggests that *Myoviridae* phages are the most common type of phage in river water, hospital, and dairy sewage, which is consistent with the findings of Alanazi et al. (2022) who used direct concentration of phages from the environment by PEG precipitation and Born et al. (2019), who used *E. coli* and other Enterobacteria hosts and, then identified *Myoviridae* as the dominant phages in sewage water [52, 54]. In contrast, Jurczak-Kurek et al. (2016), found a greater abundance of *Siphoviridae* compared to *Myoviridae* and reported the lowest abundance of *Podoviridae* in coliphages from sewage by using *E. coli* and different bacterial hosts [55]. Additionally, a study of viral communities in Lake Baikal indicated the prevalence of various families, including *Myoviridae*, *Siphoviridae*, and *Podoviridae*, supporting this study [56]. Phage isolates ST-T-K and EH-SP-TH were not identified by PCR which might be due to the absence of the target gene in the phages, and they are different phages from *Caudovirales*.

## Conclusion

The treatment of pathogenic *E. coli* is threatened by antimicrobial resistance which has become a significant issue for both medical and veterinary professionals. Lytic phages are an alternative therapy to combat antibiotic resistance. In this study, 17 phages that were lytic against multidrug-resistant diahrrgenic *E. coli* strains were discovered. The phages belonged to the order *Caudovirales*, with the family *Myoviridae*, *Siphoviridae*, and *Podoviridae*, and the genus T4 virus, T7, and T5 virus. This study provides a better understanding of the infection kinetics of seven potent phages that target *E. coli* strains, as well as their survivability under various stress conditions and growth characteristics. The phages demonstrated better growth characteristics, including short latent periods, highest burst sizes rapid growth rate, and wider host ranges, as well as thermal stability and the ability to survive in a wide range of pH levels. This information will enable researchers to determine the potential clinical application of phage-based interventions after further characterization of specific phages. These promising effects of the phage isolates against antibiotic-resistant *E. coli* have raised the possibility of their use in the biological control of bacterial infections.

## Recommendations

- This study only examined some environmental waste and one hospital sample. Dairy farm effluents are also limited. Therefore, there is a need for more research on other environmental, hospital, and agricultural/dairy farm samples.
- On the other hand, they need to be further characterized for their in vivo activity in mouse models and to assess their performance.
- Genomic factors such as virulence and drug resistance genes and the survival of these bacteriophages under different physical beside temperature and pH, and chemical conditions need to be investigated.

### Supplementary Information

**Supplementary Material 1.**

## Data Availability

All datasets that were generated or analysed during the current study were included in this paper.

## Abbreviations

*E. coli*: *Escherichia coli*
EAEC: Enteroaggregative *E. coli*
EHEC: Enterohemorrhagic *E. coli*
EPEC: Enteropathogenic *E. coli*
ETEC: Enterotoxigenic *E. coli*
IBD: Inflammatory bowel disease
PFU: Plaque forming unit
SM: Salt of magnesium
STEC: Shiga toxin-producing *E. coli*
TSA: Tryptone soya agar
TSB: Tryptone soya broth
UPEC: Uropathogenic *E. coli*
UTI: Urinary tract infection
AMR: Antimicrobial resistance
DEC: Diarrheagenic *E. coli*
SEM: Scanning electron microscopy
EOP: Efficiency of plating
MOI: Multiplicity of infection
HUS: Hemolytic uremic syndrome
DAEC: Diffusely adherent *E. coli*
WHO: World Health Organization
