# Supplementary material for: Isolation and characterization of lytic bacteriophages from various sources in Addis Ababa against antimicrobial-resistant diarrheagenic Escherichia coli strains and evaluation of their therapeutic potential
Source: BMC Infect Dis. 2024 Mar 14;24:310. doi: 10.1186/s12879-024-09152-z (PMC10938718; doi:10.1186/s12879-024-09152-z)
Supplement: Supplementary file 1 — Supplementary Material 1. [file 12879_2024_9152_MOESM1_ESM.doc]

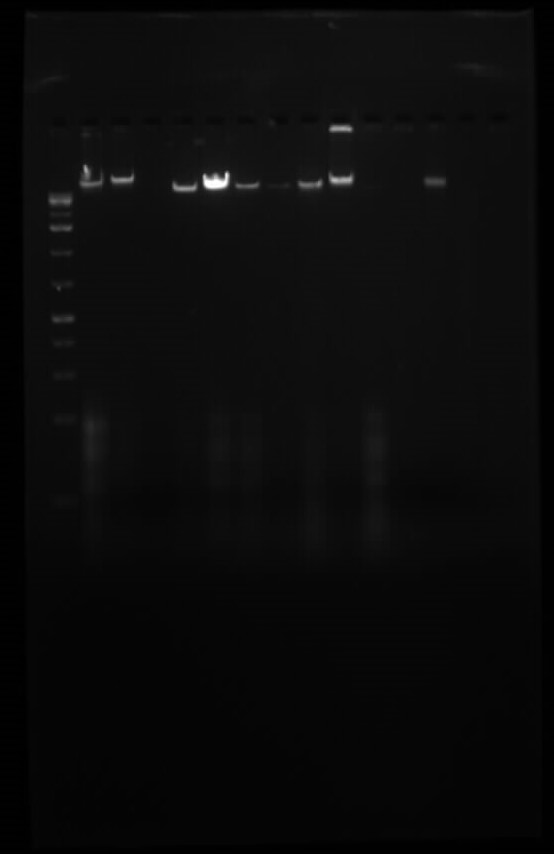


100

500

3000

1 2 3 4 5 6 7 8 9 10 11 12 13 14 15

Phage genomic DNA on 1.2% agarose gel electrophoresis: Lane1=100 bp ladder; Lane2=EP-M-A; Lane3=EP-K-K B&E2; Lane4=EH-SD-TH; Lane5=EH-B-A, A1&A2; Lane6=ST-M-A; Lane7=ST-T-K; Lane8=EI-SP-GF; Lane9=EA-T-A; Lane10=EA-SP-SMA; Lane11=EA-SD-FA; Lane12=EH-SP-TH; Lane13=ET-SD-TH; Lane14=ST-M-K and Lane15=EP-M-K.


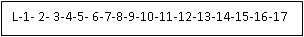

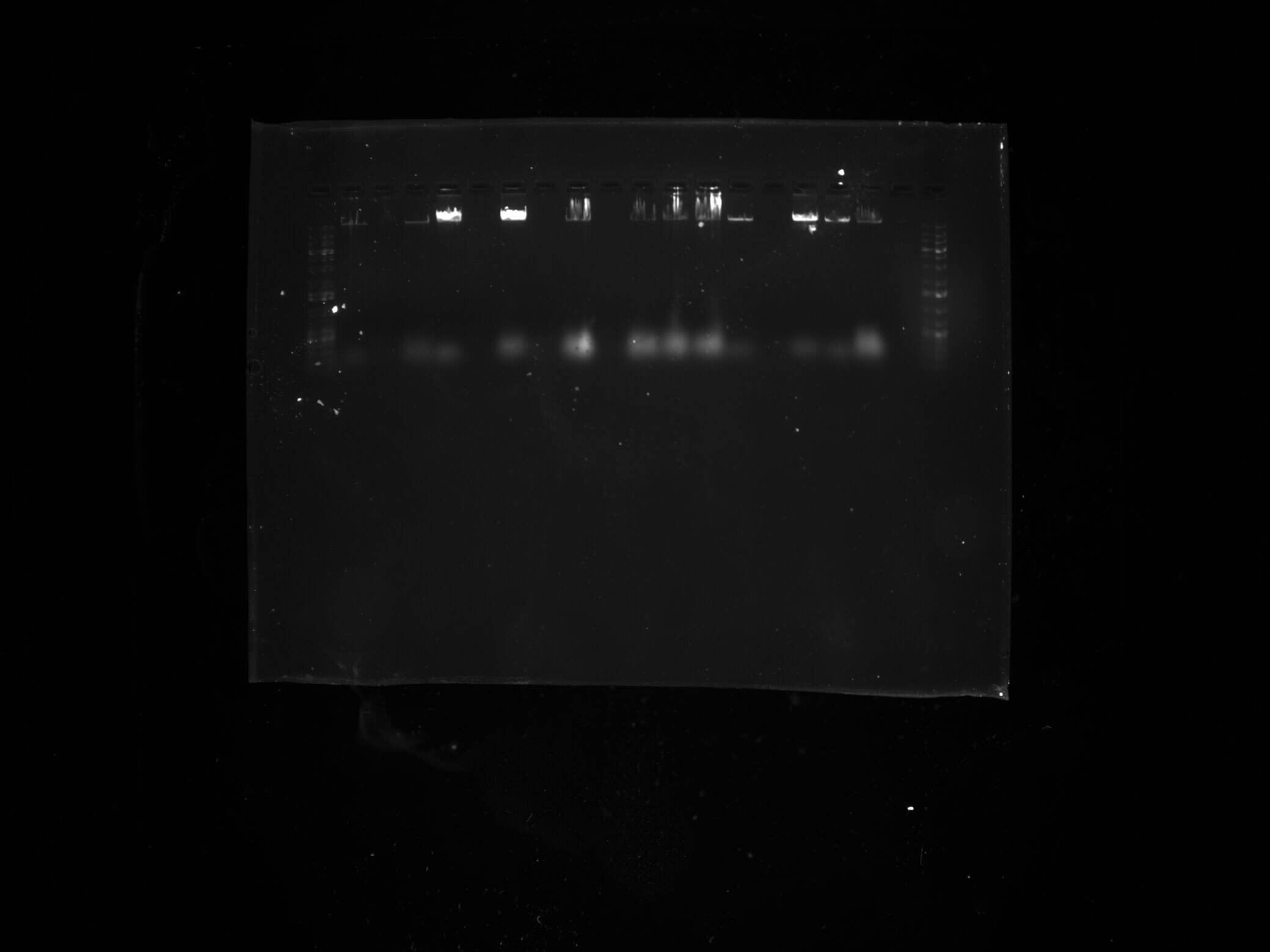

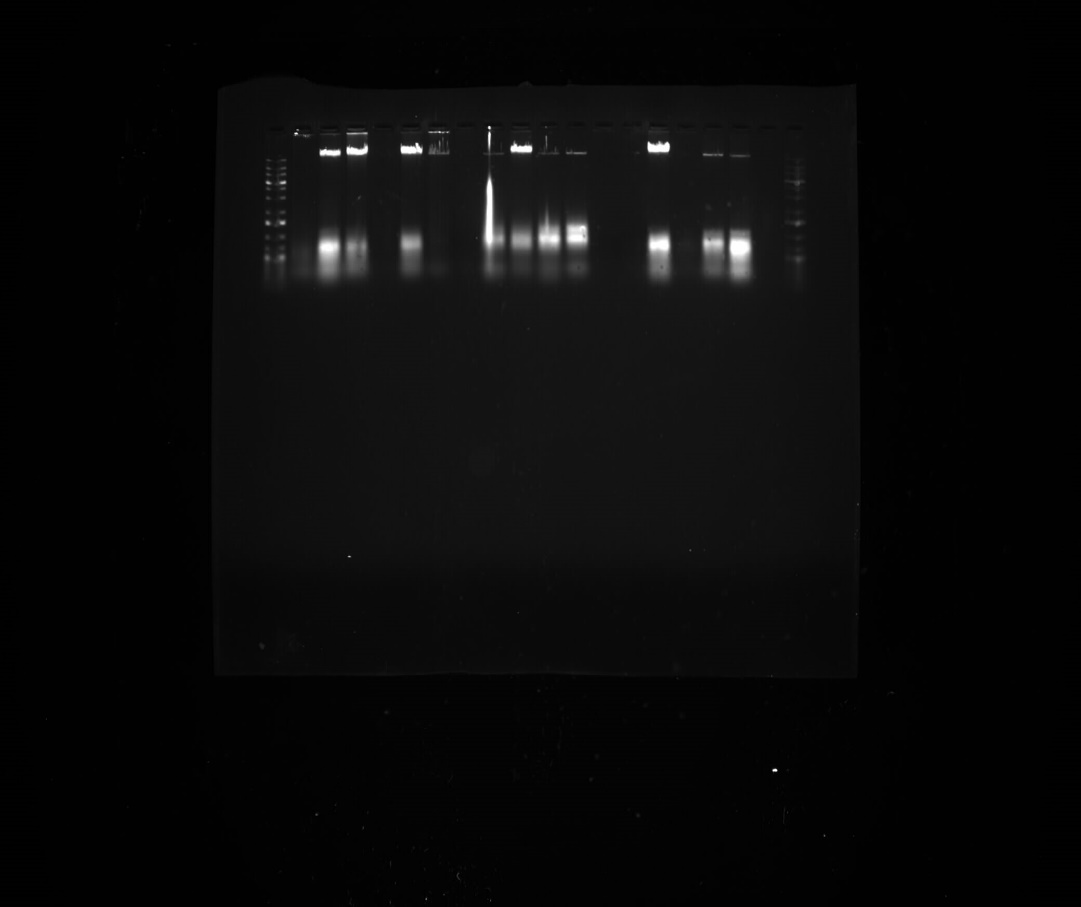


A

B

C

D

L-1- 2- 3-4-5- 6-7-8-9-10-11-12-13-14-15-16-17

1500

500

100

100

500

1500

L-1-2-3-4-5-6-7-8-9-10-11-12-13-14-15-16-17

L-1-2-3-4-5-6-7-8-9-10-11-12-13-14-15-16-17


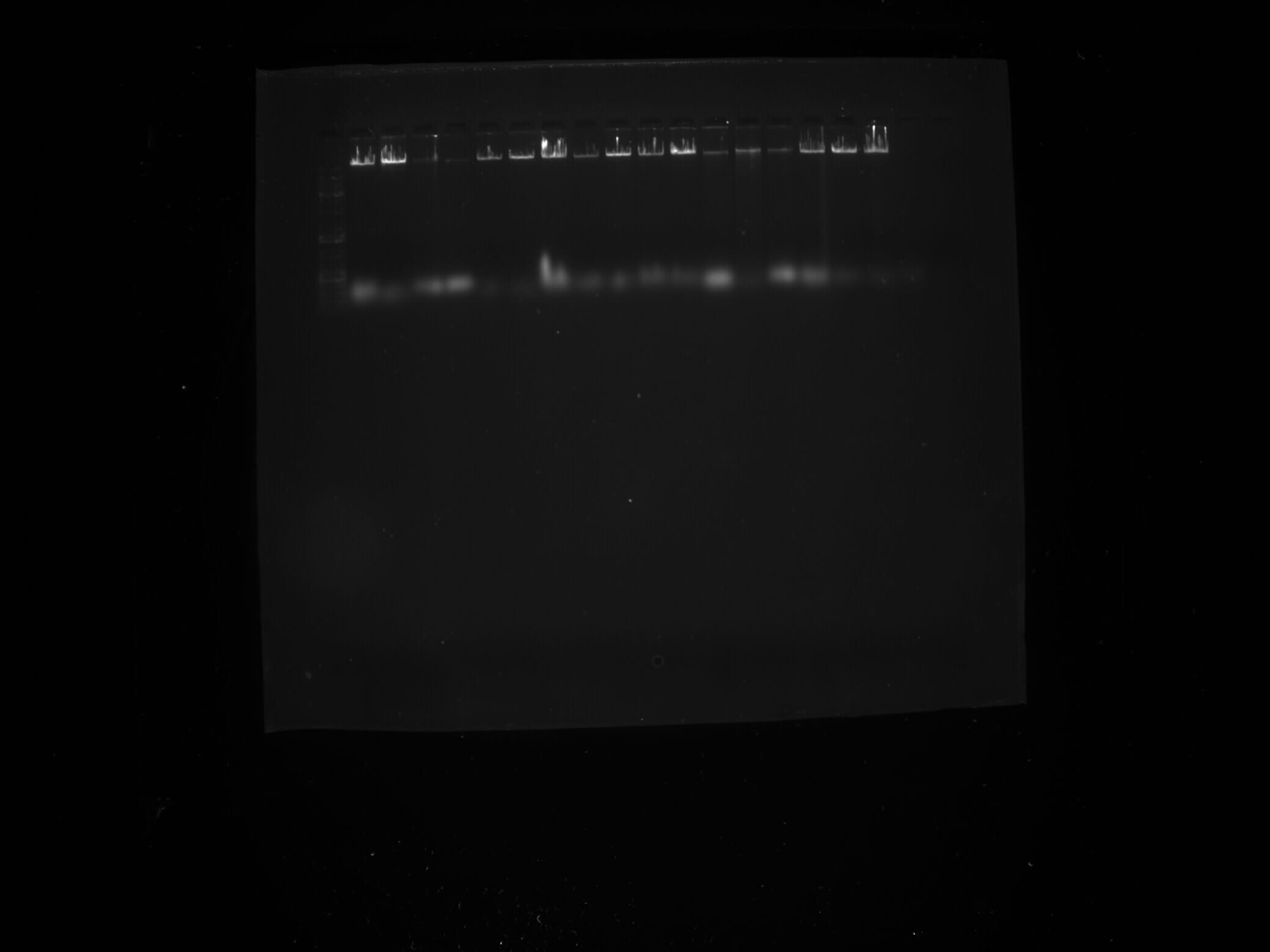

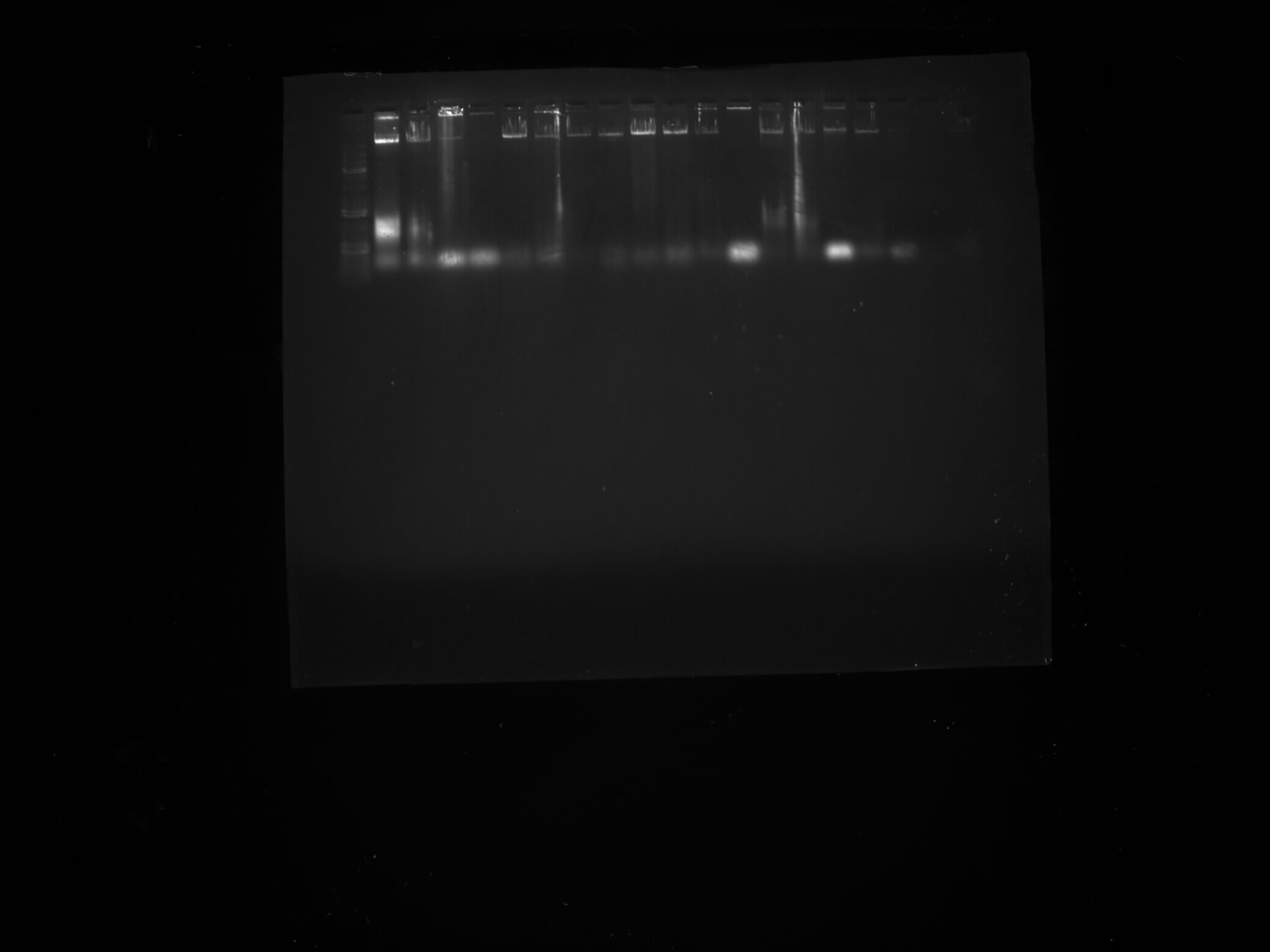


100

500

1500

100

500

1500

Comparative phage DNA extraction of 17 phage isolates to check the quality of DNA: A) phage filtrate with DNase and RNase treatment; B) filtrate with no DNase and RNase treatment; C) phage-*E. coli* mix treated with DNase, RNase, SDS, and proteinase K; D) phage-*E. coli* mix not treated by DNase, RNase, SDS, and proteinase K.

**DNA quality and quantity measured by Nanodrop**

| Phages | Concentration | Unit | A260 (Abs) | A280 (Abs) | 260/280 | Factor |
| --- | --- | --- | --- | --- | --- | --- |
| EP-M-A | 110.2 | ng/µl | 2.205 | 1.252 | 1.76 | 50 |
| EP-B-K/B | 347.6 | ng/µl | 6.953 | 3.61 | 1.93 | 50 |
| EP-B-K/EN2 | 1241.9 | ng/µl | 24.838 | 15.754 | 1.72 | 50 |
| ET-SD-TH | 387 | ng/µl | 0.779 | 0.511 | 1.67 | 50 |
| EH-SD-TH | 91.6 | ng/µl | 1.833 | 1.016 | 1.8 | 50 |
| EH-B-A/A1 | 129.1 | ng/µl | 2.583 | 1.459 | 1.77 | 50 |
| EH-B-A/A2 | 152.2 | ng/µl | 1.625 | 0.921 | 1.76 | 50 |
| ST-T-K | 751.5 | ng/µl | 15.029 | 7.394 | 2.03 | 50 |
| ST-M-A | 164.9 | ng/µl | 3.298 | 1.744 | 1.89 | 50 |
| ST-M-K | 161.9 | ng/µl | 3.239 | 1.932 | 1.73 | 50 |
| EI-SP-GF | 70.6 | ng/µl | 1.412 | 0.821 | 1.72 | 50 |
| EA-T-A | 138.3 | ng/µl | 2.765 | 1.528 | 1.81 | 50 |
| EA-SD-FA | 103.6 | ng/µl | 0.071 | 0.04 | 1.75 | 50 |
| EA-SP-SM | 164.2 | ng/µl | 3.284 | 1.852 | 1.77 | 50 |
| EA-M-A | 164 | ng/µl | 3.28 | 1.84 | 1.78 | 50 |
| EP-M-K | 190.8 | ng/µl | 1.841 | 1.032 | 1.78 | 50 |
| EH-SP-TH | 58.6 | ng/µl | 0.289 | 0.17 | 1.71 | 50 |
